# Experiences that influence how trained providers support women with breastfeeding: A systematic review of qualitative evidence

**DOI:** 10.1371/journal.pone.0275608

**Published:** 2022-10-14

**Authors:** Mary Jo Chesnel, Maria Healy, Jenny McNeill

**Affiliations:** School of Nursing and Midwifery, Medical Biology Centre, Queen’s University Belfast, Belfast, Northern Ireland; Health Social Science and Development Research Institute, NEPAL

## Abstract

**Introduction:**

There is a need to improve breastfeeding support interventions as although many are evidence-based, a sequential increase in breastfeeding rates is not evident. It is crucial to understand why the implementation of evidence-based guidelines in practice does not always translate to positive experiences for women and improve breastfeeding rates. This systematic review aims to synthesise breastfeeding support experiences of trained support providers and their impact on breastfeeding support practices.

**Methods:**

A strategy was developed to search seven databases including Medline and CINAHL and grey literature for qualitative studies. Studies eligible for inclusion reported professional and trained peer experiences of supporting women to breastfeed. PRISMA guidelines were followed and included studies were quality appraised using the CASP Qualitative Checklist. A thematic synthesis of included studies was undertaken and confidence in the review findings was assessed using the CERQual tool. The study protocol, registered in the International Prospective Register of Systematic Reviews PROSPERO registration number: CRD42020207380, has been peer reviewed and published.

**Findings:**

A total of 977 records were screened, which identified 18 studies (21 papers) eligible for inclusion comprising 368 participants. Following quality appraisal, all studies were deemed suitable for inclusion. The thematic synthesis resulted in four analytical themes: 1) A personal philosophy of breastfeeding support 2) Teamwork and tensions in practice 3) Negotiating organisational constraints and 4) Encounters with breastfeeding women. Findings demonstrated that a range of experiences influence practice, and practice evolves on continued exposure to such experiences. The potential of each experience to facilitate or inhibit breastfeeding support provision is fluid and context specific.

**Conclusions:**

Experiences, as named above, are modifiable factors contributing to the development of a philosophy of breastfeeding support based on what the provider believes works and is valuable in practice. Further research is required into the range of factors which underpin context-specific breastfeeding support practice, to improve both women’s experiences and intervention effectiveness.

## Introduction

Global breastfeeding rates fail to reach the World Health Organization (WHO) target of 50% exclusive breastfeeding until 6 months of age by 2025 [1]. This occurs despite a wealth of evidence of the health risks to mother-infant dyads of not breastfeeding [2–4], and recognition that breastfeeding is key to meeting the United Nations Sustainable Development Goals 2 and 3 for 2030 aiming to end hunger, improve nutrition and promote health [5]. Low rates of duration and exclusivity of breastfeeding are more prevalent in high-income countries (as classified by World Bank ratings) despite a range of public health interventions focusing on promoting and protecting breastfeeding [3, 6–8]. Further research into breastfeeding support interventions is required in order that the intended outcomes of increasing breastfeeding rates and positive experiences for women are more easily achieved [1, 9].

Evidence from existing reviews demonstrate that organised support from trained breastfeeding supporters, whether lay or professional, can prevent early unintended breastfeeding cessation [3, 10, 11]. Breastfeeding support is regarded as a complex intervention which involves sharing of advice and information, provision of skilled help, reassurance and increasing the mother’s confidence [12] within interpersonal interactions. Healthcare staff involved in maternal and child health services, lactation consultants and trained volunteers who have received training on how to support women to breastfeed are key to implementing effective breastfeeding support services.

Supporting the women who want to breastfeed to meet their breastfeeding goals as part of an evidence-based intervention appears to be a simple task. However, many women cease breastfeeding before they intended to do so [13–15] and some women are disappointed with the support they receive from trained providers [16–18]. Little attention is paid to the influence of factors other than evidence-based research on healthcare professional practice in relation to breastfeeding [19]. It is important to explore this knowledge gap in the context of all trained breastfeeding support providers, whether lay or professional. Indeed, the Medical Research Complex interventions framework [20] highlights the importance of the context of intervention implementation, as much as the intervention itself for successful outcomes.

Several systematic reviews have examined breastfeeding support interventions, but these studies prioritised effectiveness outcomes in terms of breastfeeding rates [3, 6, 21–23]. Such evidence is valuable but does not closely examine the underlying context of supporter behaviour. To improve provision of breastfeeding support, questions now need to be asked about contextual factors that may influence the practices of the support providers.

Relevant qualitative reviews in this area have found that, although breastfeeding support is viewed as important by both women and midwives, it can be difficult to implement the intervention effectively and compassionately. Schmied *et al*. [2011] demonstrated that women experience breastfeeding support along a continuum from a woman-centred ‘authentic presence’ to ‘disconnected encounters’ which reflect a lack of relational-based support from providers [16]. Swerts *et al*. [24] explored midwives’ perceptions of their role in support provision, reporting variance in how midwives support women with their breastfeeding [24]. These authors propose that while midwives mostly practice as a ‘technical expert’ they would prefer to practice as a ‘skilled companion’, however this requires an appropriately supportive working environment. Gaps in current knowledge persist as little is known about the factors that influence practice of the range of both healthcare professionals and trained lay breastfeeding supporters who provide routine, everyday breastfeeding support. Exploring experiences which may influence routine practice across a range of breastfeeding support provider roles is novel in relation to population and outcome, while building upon previous studies to advance the work of improving breastfeeding support provision for women. This systematic review sought to answer three research questions:

1. What is known about experiences that influence provision of breastfeeding support?
2. How do the experiences of trained breastfeeding support providers influence their breastfeeding support practices?
3. Which experiences facilitate or impede provision of breastfeeding support to women?

## Methods

PRISMA guidelines were followed in conducting this review [25]. The review protocol is registered in the International Prospective Register of Systematic Reviews PROSPERO registration number CRD42020207380 and has been published [26].

### Definitions

#### Trained breastfeeding supporters

Trained breastfeeding support providers refers to trained healthcare staff working with breastfeeding women and healthy infants/children as part of their role, and non-healthcare breastfeeding support providers such as peer breastfeeding supporters and lactation consultants who have undertaken formal accredited training. Students, untrained volunteers, healthcare staff working with sick infants/children were not included in the review as the focus was on routine breastfeeding support for mothers with healthy babies.

### Breastfeeding support

The term “breastfeeding support” in this review refers to proactive or reactive interactions between women, infants and trained breastfeeding support providers offering reassurance, praise, skilled help, problem solving, information and social support in face-to-face, group or digital settings such as social media groups, telephone calls or text messages. Support may be provided in acute hospital, maternity units, primary care, voluntary and community settings and women’s own homes. This definition is adapted from McFadden *et al*. (2017). This systematic review focuses on routine breastfeeding support in the absence of complication.

### Search strategy

A systematic search strategy was developed in collaboration with an expert subject librarian guided by a PEOT [27] question format. For the purposes of this review, Context replaces Outcome in the mnemonic as outcomes are not directly measurable in qualitative studies: Population (trained breastfeeding support providers as per study definition above), Exposure (breastfeeding and breastfeeding support provision), Context (experiences that influence breastfeeding support practices), Type of study (studies that have qualitative methods or findings).

## Study selection

### Inclusion criteria

Qualitative studies and mixed methods studies with qualitative methods and findings were included. Study findings were required to focus on trained breastfeeding support providers’ personal and professional experiences (emotions, past encounters, training, practice) in relation to breastfeeding and supporting women to breastfeed, and the influence of those experiences in providing breastfeeding support.

Reporting of ethical committee approval and evidence of data to support findings was required. The population comprised of trained breastfeeding support providers who provide routine breastfeeding support to healthy women with healthy infants in high income countries as defined by the World Bank [28]. Studies of the experiences of supporting women with breastfeeding in acute hospital, maternity units, primary care, voluntary and community settings and women’s own homes were included.

### Exclusion criteria

Mixed-methods studies that did not report qualitative findings were excluded. Studies of students, untrained volunteers and healthcare staff working with sick infants/children were not included as the focus of this review is on routine breastfeeding support for healthy mothers with healthy infants. Breastfeeding support was not considered routine when delivered to women with additional care needs [29] or delivered in a neonatal or paediatric setting. Studies with heterogeneous samples including, for example, school nurses or paediatricians were excluded if data pertaining to the experiences of trained breastfeeding support providers routinely working with women and healthy infants/children could not be isolated from dataset. Studies from low-income countries were excluded.

The search was undertaken using CINAHL +, MEDLINE ALL, Maternity and Infant Care, EMBASE, APA PsycINFO, Web of Science and Scopus databases. Reference lists of retrieved eligible studies were hand-searched for further eligible studies. An English language restriction and a methodological filter for qualitative studies was included. The reference lists of unpublished literature sourced via Open Grey and British Library Ethos were searched for relevant published studies. The search period included year 2003 –current, with the latest search completed on 16^th^ June 2021. The start year of 2003 was chosen in order to identify research undertaken following publication of the World Health Organization’s Global Strategy for Infant and Young Child Feeding (2003) [30] which advised that women exclusively breastfeed for 6 months and continue breastfeeding for two years and beyond for optimal health benefits to mother and infant. A search strategy based upon MeSH headings, related keywords and truncations was developed for each database. Boolean Operators OR and AND were used with the search terms. The search strategy is given in S1 Table of Search histories.

### Study selection procedure

Studies were selected for inclusion following a two-stage process using *Covidence* software. Findings from the searches were exported to *Covidence* via EndNote X9 reference management system enabling de-duplication of records and teamwork amongst the three reviewers. Firstly, title and abstracts were screened by the first author MJC with verification by another independent reviewer (JM or MH). Secondly, all three reviewers screened full texts independently (MJC, JM, MH).

### Data extraction

A data extraction form adapted from Healy *et al*. [31] was developed to capture information on each study’s key characteristics including study location, aim, participant demographics, methodology and method, and main findings. All text in the Findings sections of the papers, alongside verbatim quotes elsewhere in the papers, were extracted if relevant to the three research questions of the review. Meaningful sections of text were extracted that identified experiences (emotions, past encounters, training, practice) that influenced how breastfeeding support was practiced by the provider, from their personal perspective, or as observed by researchers in the included study using a discourse analysis method [40]. Data extraction was carried out by MJC and reviewed by JM & MH.

### Reflexive note

The review team are all midwives and mothers who have supported women to breastfeed as part of their career. All believe that effective breastfeeding support can enable breastfeeding which is important for the physical and psychological well-being of the mother-baby dyad. Discussion within the team was used to minimise any bias due to undue focus on study findings that aligned with personal views.

### Quality assessment

All studies were critically appraised by MJC with discussion amongst the review team. The Critical Appraisal Skills Programme (CASP) Qualitative checklist tool [32] was used to assess the quality of the studies. The COREQ tool [33] was used to assess the comprehensiveness of the reporting of study design, analysis and findings. The CERQual tool [34] was used to assess confidence in the review findings as an overall body of knowledge.

## Analysis

A systematic three-step approach to analysis was used to develop themes relevant to understanding which experiences of breastfeeding and breastfeeding support influence practice. A thematic synthesis as described by Thomas and Harden [35] was conducted. An inductive approach was chosen as the authors had no assumptions about what the dataset would reveal. Thematic synthesis facilitates an interpretation of concepts across different types of intervention, which is appropriate for breastfeeding support research because the intervention is carried out in multiple formats and settings. Thomas and Harden propose that thematic synthesis is the process of recognising cross-cutting concepts across studies, even though they may not be expressed using identical words, in order to provide new insights into policy, practice and further research [35]. Principles of thematic analysis [36–38] were used throughout coding and theme development.

Coding was conducted primarily by MJC and reviewed by the co-authors. Firstly, line-by-line coding of all relevant text extracted from the Findings sections of the papers was undertaken. Excel software was used to manage data. Resultant free codes were transcribed together with supportive verbatim text to enable ease of searching and linking to data in the studies. Descriptive themes were developed in an iterative process, moving forwards and back between suggested commonalities and disparities in the meanings of the free codes. Potential descriptive themes were discussed amongst the review team and compared across studies. Analysis of the descriptive themes led to development of four core analytical themes which reflected the synthesis of all papers in the review, agreed by all members of the review team.

## Findings

### Included studies

**Fig 1:**
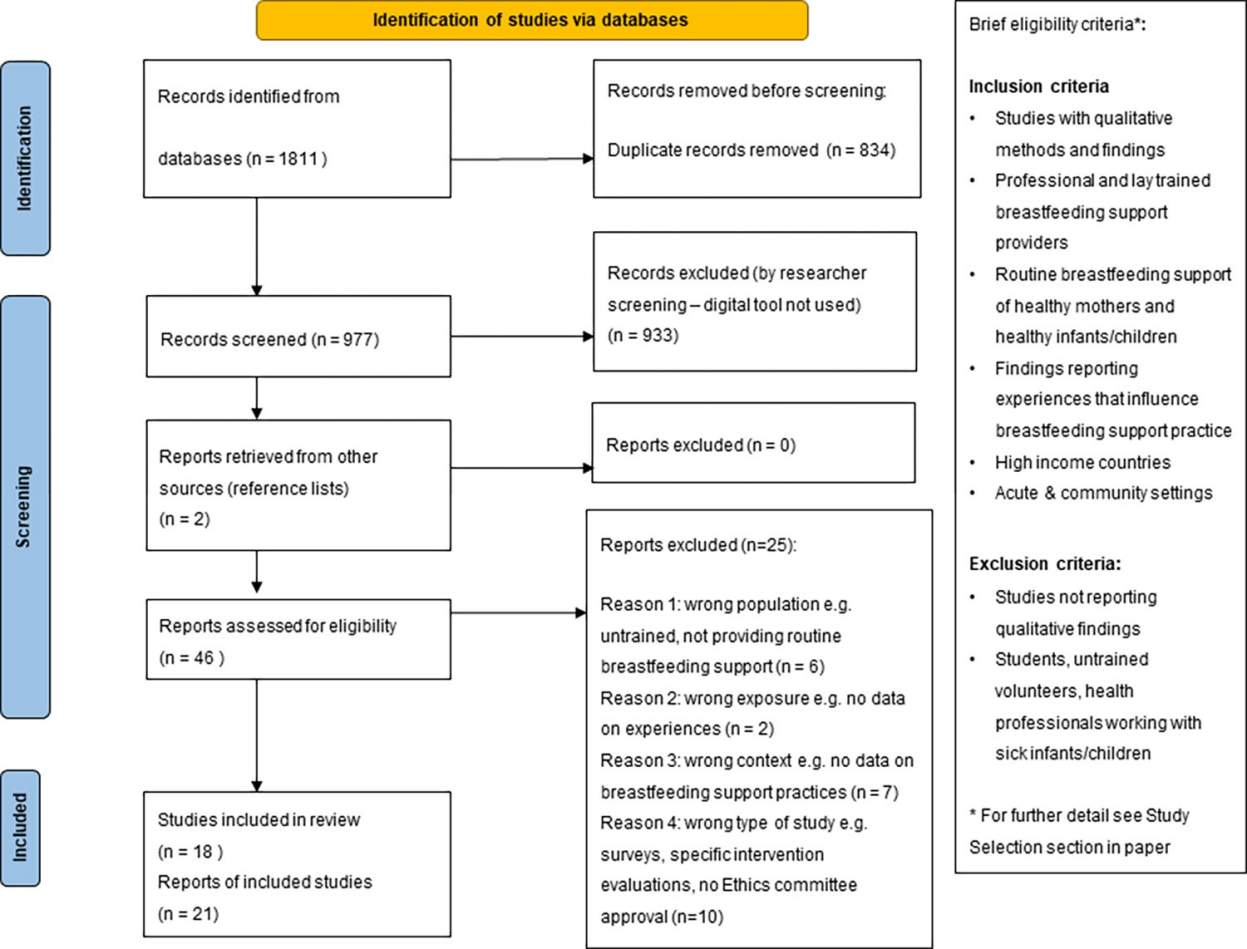
PRISMA flow chart.

The database search resulted in 1811 records, of which 834 duplicates were removed leaving 977 records for screening by title and abstract. Exclusion of 933 records following screening left 46 records for assessment, including two additional records retrieved from reference list sources. From these 46 records, 25 were excluded for reasons of wrong population (e.g. not providing routine breastfeeding support), wrong exposure (no data on breastfeeding or breastfeeding support), wrong context (no data on experiences that influence breastfeeding support practices) or wrong type of study (pilot evaluations) and 21 records were included in the review. A flow diagram adapted from the Preferred Reporting Items for Systematic Reviews and Meta-Analysis (PRISMA) guidance [25] was used to report the study selection process. Fig 1 shows the study selection process.

### Study characteristics

The dataset included 300 trained healthcare staff with identified roles: 210 midwives, 16 Health Visitors, 14 Public Health Nurses, 12 Maternal Newborn Nurses and 9 Post-partum nurses. Three studies did not specify individual role prevalence in sample groups, with one describing the sample as 20 primary healthcare professional participants comprising nurses, midwives and family physicians [39], another sample was described as 10 ward staff who were trained to support women to breastfeed including midwives, maternity care assistants and nursery nurses working on maternity ward [40] and sample group of 9 doctors and nurses was reported in a third study [41]. The trained breastfeeding support providers in non-healthcare roles in the data set comprised 56 lactation consultants and 12 peer supporters.

Eligible studies were published between 2005 and 2020 and conducted in 8 high income countries. Included papers reported qualitative designs with either interviews or focus groups apart from one Australian solely observational study [42]. Several studies used participant observation alongside interview and focus group methods. Research took place within hospitals, community primary care, community and voluntary settings and private practice. Two studies reported UNICEF Baby Friendly accreditation [43] of their setting [44, 45]. Furber’s UK based studies [46–48] reported that their setting was not Baby Friendly accredited, and all other included studies did not report Baby Friendly Accreditation status.

Data was extracted from each study comprising Title, Author, Year, Country, Study aim or research question, Practice setting, Methodology and method, Population and sample size, COREQ score and Findings (Themes/subthemes). Study characteristics and quality appraisal scores are summarized in Table 1.

**Table 1:** Study characteristics and quality rating.

Title, Author & Year	Country	Study aim/ Research question	Practice setting	Methodology & Method	Population & Sample size	COREQ score	Findings: (Themes / sub-themes)
**Lactation Consultant’s Perceived Barriers to Providing Professional Breastfeeding Support.** **Anstey *et al*. (2018)**	U.S.A.	To explore the perspectives of lactation consultants about early (prior to 4 weeks postpartum) breastfeeding problems that may lead to early weaning and identify factors that hinder the professional management of these problems.	A range of practice settings including hospitals, WIC Clinics (a nutrition program for women infants and children), private practice and pediatric offices.	Grounded theory Interviews	International Board Certified Lactation Consultants (IBCLCs) n = 30	27	Two categories of factors (direct and indirect) act as facilitators or barriers to IBCLC role enactment: **Indirect barriers** (social norms, knowledge, attitudes) **Direct occupational barriers** (institutional constraints, lack of co-ordination, poor service delivery) **Direct individual barriers** (mother’s social support, mother’s self-efficacy)
**Two sides of breastfeeding support: experiences of women and midwives.** **Bäckström *et al*. (2010)**	Sweden	To investigate women’s experiences and reflections of receiving breastfeeding support, and midwives’ experiences and reflections of giving breastfeeding support.	Hospital maternity unit	Qualitative inquiry using content analysis Interviews	Midwives n = 4	17	Individualised breastfeeding support increases confidence and satisfaction: **The unique woman** (Confirmation as a person and as a breastfeeding woman, Support to women whether breastfeeding or not.) **The sensitive confirming process** (observation and confirmation, practical and physical support) **Consistency of ongoing support** (Establish continuity, follow up)
**Liquid gold from the milk bar: Constructions of breastmilk and breastfeeding women in the language and practices of midwives.** **Burns *et al*. (2012)**	Australia	To examine how midwives represent breastmilk and construct the breastfeeding woman in their interactions with women during pregnancy and following birth.	Two hospital sites including hospital-based standard care, home-base post-natal care and continuity of care models	Qualitative Inquiry using discourse analysis Observation and interviews	Midwives n = 76	16	**Colostrum: an atomic bomb of nutrients** **Mature breastmilk as superior ‘liquid gold’** **Breastfeeding women as ‘operators’ of breastfeeding equipment**
**Mining for liquid gold: midwifery language and practices associated with early breastfeeding support.** **Burns *et al*. (2013)**	Australia	To examine the nature and impact of the language and practices of midwives when providing breastfeeding support to women in the early post-partum period.	Two hospital sites including hospital-based standard care, home-base post-natal care and continuity of care models	Qualitative inquiry using discourse analysis Observed interactions, Focus groups and interviews	Midwives n = 76 as per Burns *et al*. (2012)	22	**Mining for liquid gold** (Hands on–rights to the ‘equipment’, The tools of the trade, facilitating and fixing the equipment, the ‘expert’ midwife) **Breastfeeding–it’s not rocket science** (‘Anyone can do it’ you just need commitment, We are here if you need us, There are other ‘priorities’) **Breastfeeding is a relationship** (Getting to know the woman, Getting to know the baby, Prioritising women’s knowledge and abilities)
**"The right help at the right time": Positive constructions of peer and professional support for breastfeeding.** **Burns, E. and Schmied, V. (2017)**	Australia	To explore the similarities and differences in breastfeeding communication styles, and language and practices used, in the first month after birth, by privately practicing midwives, working in a continuity of care model, and trained breastfeeding peer support counsellors providing support at a national breastfeeding organisation’s community-based drop-in lounge.	Private midwifery group practice and a drop-in breastfeeding drop-in centre	Qualitative inquiry with discourse analysis Observed interactions	Midwives and peer support counsellors n = 9 (sample comprised of 5 privately practising midwives and 4 peer support counsellors)	23	**Breastfeeding is Normal** (Normalising breastfeeding challenges, Being a trained/professional friend) **Your body, your choices** (Facilitating autonomy, Facilitating Choice, Facilitating relationship) **The right help, at the right time**
**Public health nurses’ experiences of supporting women to breastfeed in community settings in Ireland.** **Dunne, S. and Fallon, A. (2020)**	Ireland	To explore Public Health Nurses’ experiences of supporting women to breastfeed in community settings in Ireland.	Rural and urban community settings	Descriptive qualitative inquiry Interviews	Public Health Nurses (PHN) n = 14	23	**To give them the best support** (Supporting breastfeeding is enjoyable, To support them in their decision, Letting them know we’re there) **We need the help and support as well** (I have the skills to support them, The nearest breastfeeding support group, My best support is the lactation consultant) **We’ve only a certain amount of time** (Time is a massive factor, Big time lapse, Time bothers me big time, It’s those critical times)
**A critical ethnographic study of encounters between midwives and breast-feeding women in postnatal wards in England.** **Dykes, F. (2005)**	England	To critically explore the nature of interactions between midwives and breast-feeding women within postnatal ward settings in northern England	Post-natal wards	Critical ethnography Interviews	Midwives n = 39	20	**Taking time and touching base** (Communicating temporal pressure, Routines and procedures, Disconnected encounters, Managing breastfeeding women, Rationing information)
**Breastfeeding Initiation: An in-depth qualitative analysis of the perspectives of women and midwives using Social Cognitive Theory.** **Edwards *et al*. (2018)**	Scotland	To explore women’s and midwives’ expectations, knowledge and experiences of breastfeeding initiation, including skin-to-skin contact and instinctive behaviour. (Definition of initiation is breastfeeding within the first 48 hours)	Baby Friendly Initiative accredited community, labour ward and post-natal wards	Qualitative inquiry using Social Cognitive theory for analysis Interviews	Midwives n = 18	23	**Expectations** **Knowledge** **Experiences**
**Midwives in the UK: An Exploratory Study of Providing Newborn Feeding Support for Postpartum Mothers in the Hospital** **Furber, C.M. and Thomson, A.M. (2007)**	England	To explore English midwives’ views and experiences of providing newborn feeding support.	Two non-BFI accredited maternity hospitals	Qualitative inquiry using Grounded Theory principles Interviews	Midwives n = 30	25	**Surviving Baby Feeding** (Demands on Time, Coping with newborn feeding in the hospital)
**The Emotions of integrating breastfeeding knowledge into practice for English Midwives: A qualitative study** **Furber, C.M. and Thomson, A.M. (2008a)**	England	To discover the views of English midwives in relation to their breastfeeding support role	Two non-BFI accredited maternity hospitals	Qualitative inquiry using Grounded Theory principles Interviews	Midwives n = 30 as per Furber and Thomson (2007)	21	**Surviving baby feeding: Emotionalisation of breastfeeding** (Anger in practice, Fear in practice, Sadness in practice, Happiness in practice)
**Breastfeeding practice in the UK, Midwives’ perspectives** **Furber, C.M. and Thomson, A.M. (2008b)**	England	To discover the views of English midwives in relation to their breastfeeding support role	Two non-BFI accredited maternity hospitals	Qualitative inquiry using Grounded Theory principles Interviews	Midwives n = 30 as per Furber and Thomson (2007)	24	**Surviving baby feeding: Directing Feeding** Doing well with Feeding (Communicating sensitively, Facilitating breastfeeding, Reducing conflicting advice)
**Factors influencing the sustainability of volunteer peer support for breastfeeding mothers within a hospital environment: An exploratory qualitative study** **Hopper, H. and Skirton, H. (2016)**	England	To establish the sustainability of a volunteer peer support service for new breast-feeding mothers within a hospital environment from the perspective of the volunteers and ward staff.	Hospital maternity ward	Descriptive qualitative inquiry Interviews	Maternity ward staff n = 16 (sample comprised of 6 Peer supporters and 10 ward staff who were trained to support women to breastfeed: midwives, maternity care assistants and nursery nurses working on maternity ward, role prevalence not provided).	21	**What peer supporters brought to the maternity ward** **What motivated the peer supporters** **Factors contributing to the sustainability of the service.**
**Midwives’ experiences of helping women struggling to breastfeed.** **Lawton, K. (2016)**	England	To explore midwives’ experiences of helping women who were struggling to breastfeed.	Post-natal wards and community settings	Descriptive phenomenology Interviews	Midwives n = 5	16	**Time poverty** (Breastfeeding support and time hierarchy, conditional vs unconditional motivation) **The impact on midwives on being with women** (Emotional impact on midwives, Giving permission to women to make the ‘wrong decision’, Empathising with women, How midwives are seen by women in their care) **Professional Integrity** (The questioning of personal professional credibility, Confidence in personal practice, Accountability)
**Care during Breastfeeding: Perceptions of Mothers and Health Professionals** **Lucchini-Raies *et al*. (2019)**	Chile	To know the perceptions of mothers and health professionals in relation to the care provided and received during breastfeeding at primary health care level.	Primary care level family health centres.	Qualitative Inquiry Interviews	Primary Healthcare professionals n = 20 (sample comprised of nurses, midwives and family physicians–data on role prevalence not provided).	20	**Influence of previous care and support experiences during the breastfeeding process** **Importance of the context within which care is framed** **Addressing emotions to establish trust between professionals and mothers**
**Using evidence in practice: What do health professionals really do? A study of care and support for breastfeeding women in primary care** **Marshall *et al*. (2006)**	England	To examine the use of knowledge, and in particular, of evidence derived from research, in interactions between midwives or health visitors and breastfeeding women	Community settings in rural, suburban and urban settings	Ethnography Observations and interviews	Midwives and Health visitors n = 18 (sample comprised of 9 community midwives, 9 health visitors)	17	**Professional experience** **Formal courses and education** **Knowledge from research and Evidence Based Practice** **Knowledge from policies and guidelines** **Using colleagues as a source of knowledge** **Personal experience** **Knowledge use in practice**
**Maternal-newborn nurses’ experiences of inconsistent professional breastfeeding support** **Nelson (2007)**	U.S.A.	To describe the meaning and significance of the common, essential elements of inconsistent professional breastfeeding support as revealed through the experiences of maternal-newborn nurses in a hospital setting	nursery post-partum, labour and delivery settings	Existential (descriptive) phenomenology Interviews	Maternal newborn nurses n = 12	21	**Inconsistencies still exist but things are changing** **A need for "buy in"** **There is no escaping personal experience** **What works for one does not work for all** **Time impacts on recommendations** **A privileged vantage point** **My job" what it is and what it is not "** **After all, breastfeeding is maternal choice**
**Exploring how IBCLCs manage ethical dilemmas: a qualitative study** **Noel-Weiss *et al*. (2012)**	U.S.A. and Canada	To explore ethical dilemmas experienced by IBCLCs, especially, how they manage such dilemmas.	Hospitals, private practice, public health and community settings	Qualitative inquiry Interviews	IBCLCs n = 7	23	**Staying mother-centred** (Recognising an ethical dilemma or issue, Identifying context, Determining the IBCLC choices, Strategies used, Results and choices the mother made, Follow-up and actions to avoid similar situation)
**The supporting role of the midwife during the first 14 days of breastfeeding: A descriptive qualitative study in maternity wards and primary healthcare.** **Swerts *et al*. (2019)**	Belgium	To gain an in-depth understanding of the supporting role of midwives in breastfeeding support during the first two weeks after birth and compare it to the needs of the breastfeeding women.	2 Hospital settings (one with BFI accreditation and one without) and home settings in public and private midwifery care.	Qualitative inquiry Observation and focus groups	Midwives n = 21 (observations, of which n = 10 participated in subsequent focus group)	23	Focus of the midwife Approach and expectations Hands on–hands off Observing Informing Coaching and supporting Relationship between midwife and mother
**Barriers to breastfeeding: a qualitative study of the views of health professionals and lay counsellors** **Tennant *et al*. (2006)**	England	To ascertain the views of staff about their breastfeeding support skills, and their own attitudes to supporting breastfeeding mothers, to inform the development of a training intervention that would address these attitudes as well as build knowledge.	General Practice, Health clinics and National Childbirth Trust (NCT) lay counsellor settings	Qualitative Inquiry Interviews	Health visitors, midwives and NCT breastfeeding counsellors n = 12 (sample comprised of 7 health visitors, 3 midwives and 2 NCT breastfeeding counsellors)	20	**Helping women make a decision about feeding** **Factors shaping professional practice** **Access to training**
**Medicalizing to demedicalize: Lactation consultants and the (de)medicalization of breastfeeding** **Torres, J.M. (2014)**	U.S.A.	Research questions: 1) To what extent, and in what ways, do lactation consultants work towards demedicalization? 2) How do lactation consultants balance demedicalization with their role as the clinical managers of breastfeeding?	Hospital inpatient and outpatient settings	Ethnography Observation and interviews	IBCLCs and doctors, midwives and nurses n = 28 (sample comprised of 19 IBCLCs and 9 healthcare professionals who were doctors and nurses–data on role prevalence not provided).	16	**Demedicalizing breastfeeding** (pathology, technology and medical control) **Medicalizing breastfeeding** **Medicalizing to demedicalize**
**Personal Infant Feeding Experiences of Postpartum Nurses Affect How They Provide Breastfeeding Support.** **Wright, A.I. and Hurst, H.M. (2018)**	U.S.A.	To describe the experiences of postpartum nurses when feeding their own infants and explore how these experiences influence the breastfeeding support they provide to new mothers.	Hospital postpartum unit	Qualitative inquiry Interviews	Postpartum nurses n = 9	25	**Personal infant feeding experiences** (The decision to breastfeed was clear, Early breastfeeding was challenging, Breastfeeding after the maternity hospitalization was characterised by love or struggle, Coming to terms with the breastfeeding experience was easy for some and difficult for others)

### Critical appraisal

The Critical Appraisal Skills Programme (CASP) Qualitative checklist [32] was used to initially assess the quality of each study. A numerical score for quality was not assigned, rather, the tool prompted focused reading of papers regarding potential methodological limitations. The quality of reporting in the studies ranged from a score of 16 to 27 out of a possible 32 items in the COREQ checklist. Most studies did not report on researcher reflexivity. Description of analytical methods was limited in five of the studies. Information from the COREQ and CASP assessments enabled trustworthiness in the findings of each study to be gauged, and informed subsequent assessment of confidence in the findings using the Confidence in the Evidence from Reviews of Qualitative Research tool (CERQual) [49–53]. Overall, the strengths of the studies lay in the congruence of research aims and objectives with the study design.

### Themes

This review aimed to answer three research questions, asking what is known about experiences that influence provision of breastfeeding support, how such experiences influence breastfeeding support practices, and which experiences facilitate or impede provision of breastfeeding support to women. The findings of this review suggest that a range of prior and current experiences relating to breastfeeding and breastfeeding support provision, reported here as analytical themes, are very likely to contribute to breastfeeding support practices, either facilitating evidence-based compassionate care or hindering such provision. Synthesis of the data resulted in the generation of 85 free codes, eleven descriptive themes and four analytical themes supported by summary statements (Table 2). The prevalence of the descriptive themes and analytical themes in the papers is reported in S2 Table. Confidence in the review findings was assessed using the CERQual tool [34]. There was high confidence in three of the descriptive themes. Confidence was downgraded to moderate (five descriptive themes) or low (three descriptive themes) when there was concern about any of the four components of the CERQual assessment [34] methodological limitations, coherence, adequacy of data and relevance as reported in S3 Table. The analytical themes are titled *1) A personal philosophy of breastfeeding support 2) Teamwork and tensions in practice 3) Negotiating organisational constraints* and *4) Encounters with breastfeeding women*.

**Table 2:** Theme development.

Examples of free codes that contribute to descriptive themes	Descriptive themes *with associated CERQual assessment of confidence*	Analytical themes *and summary statements*
Personal experience Beliefs about breastfeeding Prioritising breastfeeding Sources of information Feedback	Personal breastfeeding experience *(high confidence)* Belief in the value and process of breastfeeding *(high confidence)* Knowledge for practice *(moderate confidence)*	**A personal philosophy of breastfeeding support** *The experience of having a personal philosophy of breastfeeding support promotes individual practice styles*
Referral Actions of others Undermining others Inconsistent advice	Collaboration *(moderate confidence)* Inconsistency in care and advice *(moderate confidence)* Opinions of others *(low confidence)*	**Teamwork and tensions in practice** *The experience of disconnection from or collaboration with colleagues undermines or progresses breastfeeding support practice*
Time constraints Resource to enact role Priority of breastfeeding support	Time and resources *(high confidence)* Organisational values *(moderate confidence)* Expectation of role *(low confidence)*	**Negotiating organisational constraints** *The experience of negotiating organisational expectations and constraints impacts role-enactment*
Mother’s confidence Mother’s commitment Influences on mother Communication with mothers Trusting relationship	Perceptions of mother’s breastfeeding reality *(low confidence)* Relationship and communication *(moderate confidence)*	**Encounters with breastfeeding women** *The experience of interacting with breastfeeding women fosters a tailoring of support to women’s perceived needs*

For brevity, exemplar quotes of data are presented below and further supporting quotes for each descriptive theme are found in the CERQual Evidence table (S3 Table).

### A personal philosophy of breastfeeding support

A personal philosophy of breastfeeding support was the most dominant analytical theme of the review. It is comprised of three descriptive themes, further detailed in subsequent sections, which show that breastfeeding support is delivered according to the providers prior experiences, established beliefs and preferences in applying knowledge for practice from various sources. Participants in all studies spoke of experiences which were developed into 3 interlinked descriptive themes to form a personal philosophy of breastfeeding support: *Personal breastfeeding experience* [39, 40, 42, 44, 45, 54–60] rated by CERQual assessment as having a high level of confidence in the finding, *Belief in the value and process of breastfeeding* [39–42, 45, 58, 60–63] which also had high confidence as a finding, and *Knowledge for practice* [39, 40, 42, 44, 45, 47, 48, 56, 57, 59–64] in which there was moderate confidence in the finding.

#### Personal breastfeeding experience

Participants frequently described having a personal understanding of ‘what matters’ and ‘how-to’ in breastfeeding support, borne of prior experience. Both positive and negative personal breastfeeding experiences increased empathy for breastfeeding women. Participants tried to help women avoid the physical discomfort or psychological distress that they had encountered personally, either encouraging women that breastfeeding difficulties were surmountable, or not. Participants spoke of giving women permission to stop breastfeeding or supplement with formula, or spending time and offering breastfeeding tips and tricks learned from personal experience: *“Before I had [my baby] it was like, “Oh, you want formula? Okay, I’ll go get it.” It was more patient satisfaction versus “what was your intention when you came in? You wanted to breastfeed. I will help you become successful in breastfeeding” [60]* (Post-partum nurse in a hospital setting). The above quote illustrates how personal experiences of breastfeeding could both inform and transform practice.

#### Belief in the value and process of breastfeeding

There was little diversity across the studies in terms of belief in the value and process of breastfeeding. Most participants spoke of the importance and value of breastmilk and/or breastfeeding to a mother/baby dyad:*“…midwives focused on ensuring that the infant had sufficient access to ‘liquid gold’*. *Midwives drew on their ‘expert’ knowledge to introduce a range of techniques and technology to ensure that the infant received breastmilk”* [61]. Although belief in the value of breastfeeding was widely represented in the data, beliefs about the skills involved in breastfeeding varied. Some participants believed that breastfeeding was a natural skill that anyone could do if they were prepared to work at it, whereas others saw breastfeeding as a technical skill that women had to be taught.

#### Knowledge for practice

Participants spoke of the personal and vicarious learning experiences that informed their practice. Experiential knowledge, attending training courses, following guidelines and policies, engagement with research and learning from colleagues were all identified as influential. Participants selected what information, gained from experience, they felt may be useful in different situations. This was then tested against knowledge from prior breastfeeding support episodes and input from colleagues. However, working in accordance with guidelines resulted in some participants expecting to physically intervene in breastfeeding support: *‘…midwives described an expectation of giving “hands on” help with attachment in the labour ward… This may arise in part from BFI guidelines [which recommend skin-to-skin and breastfeeding within the first hour of birth]’* [45]. The experience of working to guidelines influenced the physical approach to support.

### Teamwork and tensions in practice

Data from 14 papers contributed to this analytical theme, comprised of 3 descriptive themes: *Collaboration* [48, 54, 55, 64] in which there was moderate confidence in the finding, *Inconsistency in support and advice* [45, 48, 54, 55, 58, 64] in which there was also moderate confidence and *Opinions of others* [44, 45, 47, 55, 56, 61] in which the authors had low confidence.

#### Collaboration

Positive collaborative experiences included effective communication with others involved in breastfeeding support, a visible and effective referral system in place and respectful relationships between providers. Good teamwork with sharing of knowledge facilitated breastfeeding support provision. Nevertheless, participants often reported problems with collaboration and referral, either with the availability of staff such as lactation consultants only working certain shifts, or with other support providers waiting too long before referring a mother to expert help. The following quote highlights the frustration felt by a lactation consultant when late referrals impeded the ability to provide effective support to women experiencing breastfeeding difficulties: *“they’re usually train wrecks”* [54]. This reveals a sense of despair that other breastfeeding providers do not refer women in a timely manner so that early problems may be resolved more easily.

#### Inconsistency in support and advice

Inconsistency in support and advice was a common theme. Participants undertake damage limitation techniques to reassure the woman that what they perceive as inconsistent advice is a normal aspect of the breastfeeding learning journey. Managing conflicting and potentially damaging input from others resulted in trying to achieve a balance between offering effective support and not wishing to undermine a mother’s belief in other breastfeeding supporters and healthcare providers. This was an uncomfortable experience for some participants.

#### Opinions of others

Differing philosophies of breastfeeding support between providers impeded effective provision. Participants experienced intimidation and disapproval from some colleagues if they devoted time to breastfeeding support. Peer opinion also facilitated a more physical direct approach: ‘*…being able to competently attach an infant*, *often referred to as ‘having the knack’*, *was a highly prized and sought-after skill that afforded some midwives a sense of status within their professional peer group’* [61]. Experiences of perceived credibility with colleagues therefore influenced how breastfeeding support was provided.

### Negotiating organisational constraints

Participants in 11 papers contributed to the 3 descriptive themes informing this overall analytical theme: *Time and resources* [40, 42, 44, 45, 47, 48, 54–56, 58, 61, 62] which had high confidence in the findings, *Organisational values* [45, 47, 54, 55] in which there was moderate confidence in the findings and *Expectation of role* [41, 45, 47, 56, 58] in which the authors had low confidence as a finding when assessed using CERQual.

#### Time and resources

The impact of being under-resourced in terms of time pressures and staff shortages prevented participants from providing optimal breastfeeding support. Participants spoke about stepping in to physically attach babies to the breast in the hope that it would save time and free them to attend to other clinical duties. In acute settings it was common for staff to default to such time-saving approaches to breastfeeding support even when there was no pressure on resources. Conversely, some participants who independently organise and control their workload, for example privately practising midwives, were able to spend time to optimise the support given to breastfeeding women.

#### Organisational values

The low priority given to breastfeeding as a maternity care issue was identified as something which hinders good breastfeeding support practices. One midwife in a hospital setting spoke of decision-making in terms of breastfeeding support and available resources: *“Sometimes you have to say I’ll send a midwife out*, *but you know that’s a resource that is precious*. *All you can do is make sure they’ve got a visit the next day if it’s the middle of the night*. *[Pause] But that’s a long gap and that’s not her answer at that time”* [47] (midwife providing telephone support on night shift). When the organisation is under-resourced breastfeeding support is not always viewed as a worthy recipient of an organisation’s resources.

#### Expectation of role

This descriptive theme relates to the complex issue of being a breastfeeding supporter who is on one hand tasked with promoting and protecting breastfeeding, and on the other, being respectful of women’s choices. One maternal newborn nurse in a postpartum unit reported “*Breastfeeding shouldn’t be a hard sell*…*I mean my job is not to push somebody”* [58] demonstrating that self-perception of role led to decisions about how much encouragement to give to women.

### Encounters with breastfeeding women

Participants in all but one of the papers contributed to this analytical theme relating to interactions with breastfeeding women, comprised of 2 descriptive themes: *Perceptions of women’s breastfeeding reality* [42, 44, 45, 47, 48, 54–56, 58, 61–64] in which there was low confidence in the finding, and *Relationship and communication* [39, 41, 42, 44, 47, 48, 54, 56–58, 60–65] in which there was moderate confidence in the finding. Encounters with women were opportunities to assess the woman’s individual needs and also her commitment to breastfed. This information then influenced how support was provided in terms of motivation to provide support and time spent with women. However, capturing the woman’s wishes required open communication and some connection or relationship to be established.

#### Perceptions of mothers breastfeeding reality

Perceptions of the mother’s motivation and capability in relation to achieving breastfeeding goals were early signs used by the provider to gauge how to provide support. Some participants spoke of the impact of a mother’s commitment on their own motivation in practice, for example midwives in a hospital setting: *“the women have to have some sort of commitment as well*. *If they don’t have that commitment*, *then there’s no point in us busting our gut to do it*, *either*” [61]. Thus some participants made value judgements on breastfeeding women to assess whether it was a worthwhile priority for care. This was not reported in settings were women actively sought support, for example in drop-in clinics or during consultation with a lactation consultant.

#### Relationships and communication

Some participants spoke about their sensitivity to women’s views on their practice which influenced how information was conveyed: *‘Sometimes you know you’ve to be careful that it doesn’t come across very dictatorship’ [55].* They were aware of the potential vulnerability of women and the need to communicate sensitively. Relaxed and companionable relationships were used to dismantle a novice/expert dynamic in breastfeeding support in some community settings and private practice. Encounters that respected breastfeeding women’s autonomy facilitated breastfeeding support that did not involve touching the woman or “doing for” her.

### Thematic synthesis

**Fig 2:**
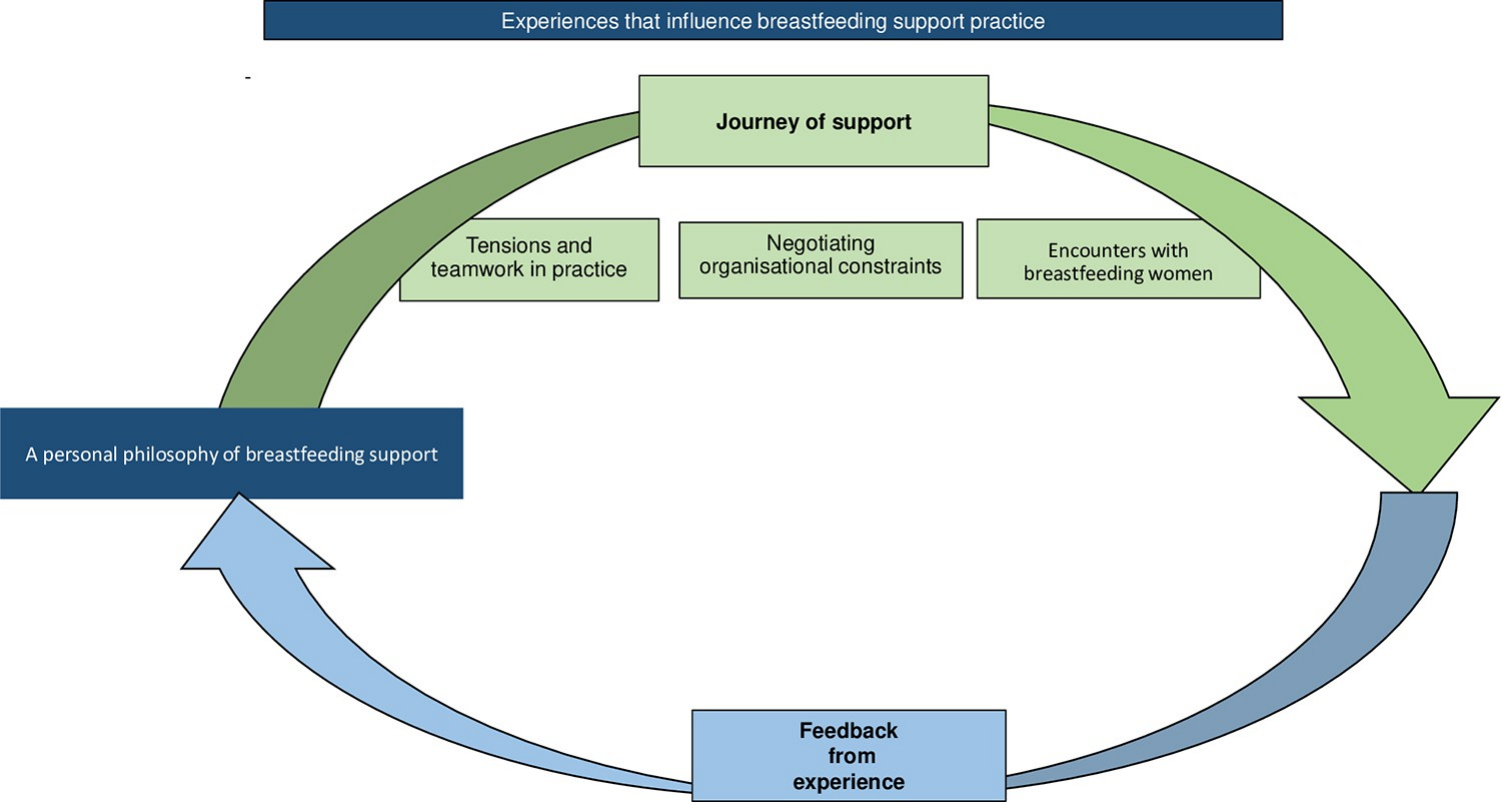
Thematic synthesis.

The thematic synthesis resulting from the interpretation of the four analytical themes demonstrates that trained breastfeeding support providers carry a philosophy of breastfeeding support with them as they start out on a journey to provide support. Findings indicate that providers have an established philosophy of support informed by personal and vicarious breastfeeding experiences, a belief (or not) in the value of breastfeeding, and preferences for “what works” in breastfeeding support practice. The philosophy of breastfeeding support develops over time with accumulation of experience. Training may shape an initial philosophy derived from participants’ personal and socio-cultural reference points, but it is further developed by the experiential knowledge from practice and any new personal and vicarious breastfeeding support experiences. Reflection on and feedback from experience therefore inform the philosophy of support in a feedback system, either re-enforcing existing beliefs or introducing new information for future practice. The philosophy of support can be suppressed or enabled depending on the immediate context in which the support is being provided. There is fluidity in how the experiences affect eventual support provision, as supporting individual women in specific settings is dynamic and responsive to the overall context within which care is given. The Thematic Synthesis is represented in Fig 2.

## Discussion

Three research questions were posed in this review; what is known about experiences that influence breastfeeding support practices, how those experiences are influential and how experiences impede or facilitate support provision. This section of the paper will initially explore how the research questions have been addressed and continue with a discussion of results in the context of the wider evidence base. In our view this is the first qualitative evidence synthesis of research reporting on breastfeeding and breastfeeding support related experiences that influence the practice of a range of trained breastfeeding support providers. From the inclusion of 21 papers in the systematic review four analytical themes were generated which illustrate experiences that influence how breastfeeding support is valued, prioritised, and delivered in practice: *A personal philosophy of breastfeeding support*, *Teamwork and tensions in practice*, *Negotiating organisational constraints*, and *Encounters with breastfeeding women*. Findings reveal that exposure to and engagement with these diverse experiences cut across both professional and peer role-types, and apply across maternity and family care settings. Regardless of training undertaken, the life history of the provider and the context bound nature of breastfeeding support practice influence how providers enact their role. A philosophy of breastfeeding support develops over time. Positive and resilient philosophies of breastfeeding support are informed by positive personal experiences, belief in the value and process of breastfeeding, service models that enable open communication with women, adequate resourcing in terms of workforce and time to spend with women, and appropriate information sources to inform practice.

The second research question focused on how experiences influence practice. Findings indicate that a range of experiences influence provider motivation and the approach to support. Decisions about whether to step away to leave a woman to learn about breastfeeding herself, whether to stay with a woman and spend time discussing her breastfeeding, or whether to take over and intervene are underpinned by the provider’s own philosophy of support, their experience of colleagues breastfeeding knowledge, skills and attitudes, the resourcing of services and experiences of prior interactions with breastfeeding women. These experiences ebb and flow in their contribution to practice which can both hinder and facilitate breastfeeding support provision. Supporting a particular mother, with particular colleagues, in a particular organisation influences behavioural aspects of how support is provided.

Finally, the review sought to explore which experiences facilitate or impede support provision. Having a belief in the value of both breastmilk and the breastfeeding relationship, having positive personal experiences including having overcome difficulties, and use of a wide range of knowledge sources (including women’s feedback about support received) appear to facilitate breastfeeding support provision. Honest, trusting relationships with women, respectful collegiate relations, peer learning and providing support in well-resourced organisations which visibly value breastfeeding also facilitate compassionate evidence-based breastfeeding support. Negative breastfeeding experiences or no breastfeeding experience can result in doubt about the value of breastmilk and the breastfeeding relationship, particularly when mothers are struggling with feeding challenges. Vicarious experiences of colleagues’ behaviours may re-enforce outdated or insensitive practices. Funding cuts to breastfeeding support services and a lack of training opportunities beyond a basic level, send a signal that breastfeeding support is not valued by an organisation. Lack of personal and professional reference points about successful breastfeeding (including no feedback from women) can result from fragmented care. An implication of such lack of feedback is that there is an absence of recognition of “what works” in practice, limiting growth and progression in the philosophy of breastfeeding support.

This review extends our understanding of why breastfeeding support provision can tend toward non-standardised, inequitable delivery and why women are reporting some dissatisfaction with their breastfeeding support despite providers being trained for the role. New insights into the importance of personal and vicarious experiences of breastfeeding and breastfeeding support have emerged that require us to think differently about the influence of provider’s ongoing experiences of breastfeeding and breastfeeding support, and the communities of practice in which women are supported. Findings complement prior studies reporting low confidence and learning deficits in breastfeeding support providers [66, 67]. Furthermore, findings suggest that attention be given to personal and organisational factors in addition to education when considering how best to support women to breastfeed. Prior research has reported that effective support intervention implementation is complex and culturally specific [68, 69]. This systematic review enhances our understanding of the complexity in implementing breastfeeding support interventions. The principal implication is that an individual’s approach to breastfeeding support is shaped by personal preferences, which are influenced by organisational culture, wider society and the breastfeeding support education that they receive.

The key message from the first theme *A personal philosophy of breastfeeding support* is that provider approach and behaviour during breastfeeding support encounters is grounded in how they personally make sense of breastfeeding. Exposure to skilled sensitive breastfeeding support in practice, evidence-based education, and opportunities to reframe negative experiences through reflection are required to develop a positive philosophy of breastfeeding support. Prior and current experiences of breastfeeding and breastfeeding support underpin what is valued in sharing information and teaching practical techniques. Various practice styles have been identified in earlier research into the woman’s perspective of breastfeeding support, for example perception of the encounter by women as authentic or disconnected [16] or the provider acting as a skilled companion or a technical expert [24]. The findings of this systematic review give insight into such variations. Providers tended to either step in and intervene, distance themselves so that the woman learns about her own breastfeeding by herself, or be present with a woman to discuss her breastfeeding and provide support in a partnership approach. Time spent with women, the type of information and encouragement given, and behaviour during skilled help depend on both the provider’s philosophy of support, and the ability of the provider to practice according to their philosophy within their particular workplace or volunteer setting.

The *Teamwork and tensions in practice* theme highlighted the importance of collegiate relations in practice. Development of communities of breastfeeding practice with skilled and motivated colleagues enhances teamwork and minimises tensions. Positive collaboration, efficient referral systems amongst support providers and the experience of being part of a knowledgeable team that values breastfeeding facilitates the creation of ad-hoc learning opportunities, easy access to breastfeeding expertise, and freedom to dedicate time to breastfeeding support without disapproval from colleagues. Lactation consultants were identified as a good resource for clinical staff when they were integrated into healthcare structures. These findings support the idea that informal learning and role modelling from experts in practice across multisector breastfeeding support settings are useful experiences that develop confidence in breastfeeding support skills [70]. Such opportunities should be recognised for their value in order to support the sustainability of this model [71]. The challenge now is to deliver effective breastfeeding services which foster peer learning in practice from valued experts, ease of referral to specialist breastfeeding support and everyday exposure to positive breastfeeding support provision from colleagues.

Attention must also focus on the influence of environments with tensions in practice due to differences in individual philosophies of breastfeeding support. The findings demonstrate that support providers can feel pressure from colleagues to underplay their breastfeeding support role or deviate from the evidence-base to free up time for other duties. This adds to existing evidence that experiencing colleagues outdated practice [72] and disjointed services [73] impede effective breastfeeding support provision. There is a need for ongoing breastfeeding education in practice with opportunity for providers to reflect on and in practice together. Pragmatically, multi-sector case study presentations could be scheduled as part of continuing breastfeeding education, with collaborative learning and exposure to positive examples of how skilled professional and peer support can enable women to overcome breastfeeding challenges. The importance of multisector collaborative working supports a recent qualitative evidence review into experiences of breastfeeding peer support [74] which proposed that tension between peer supporters and health professionals can be overcome through building trusted relationships in integrated services. This systematic review extends knowledge about the benefits of multisector working as experiencing the enthusiasm and skill of a variety of motivated providers allows others to develop their own confidence and skill in practice, benefitting women and ensuring that integrity in breastfeeding support services is upheld.

The third theme *Negotiating organisational constraints* illustrates how underfunding and the low prioritisation of breastfeeding support services limits role-enactment. Full implementation and funding of evidence-based breastfeeding support interventions is imperative for providers to experience services that value breastfeeding. Consistent with the literature, participants from studies included in this review reported that working under organisational constraints, especially time pressures, impedes practice [75–77]. The experience of providing support in well-funded services with adequate providers available enables provision of meaningful breastfeeding support because time, training and informal learning opportunities are available. Findings are consistent with components of the Breastfeeding Gear Model [78]. This tool is a multi-level and multi-sector approach to scaling up intervention implementation and identifies resourcing and political will as indispensable factors that enable sustainable and effective evidence based breastfeeding support intervention implementation [69]. If providers experience under-resourcing they make decisions about care priorities and breastfeeding support is not prioritised. These findings support the work of other studies linking understaffing and lack of time with lack of breastfeeding support provision [75, 79, 80] and women’s perceptions that providers are unable to provide breastfeeding support due to a lack of time and resources within healthcare organisations [75, 77]. Funding cuts to breastfeeding support services are therefore not only detrimental to women’s breastfeeding experiences [81] but also detrimental to provider development. Such cuts to services impede exposure to breastfeeding that could influence a provider’s philosophy of support. In addition, when organisations do not prioritise breastfeeding, the lack of breastfeeding education beyond a basic level limits new knowledge development, sending a signal that breastfeeding support is not a priority and providers lose motivation to enact their role.

The final theme *Encounters with breastfeeding women* describes how the experience of interacting with breastfeeding women enables support to be tailored to women’s perceived needs. Models of relational continuity in breastfeeding support should be promoted as these foster individualised breastfeeding support provision. It is known that making a connection with women, and communicating openly about breastfeeding goals, enables effective breastfeeding support provision [82, 83]. Overall this systematic review strengthens the idea that support which involves insight into the woman’s needs and preferences about breastfeeding can be facilitated by relational continuity of carer [83, 84]. Relationship has previously been highlighted in the literature as an important component of breastfeeding support [76, 85]. These findings also concur with research demonstrating the complexities in providing individualised breastfeeding support [86] because providers want to support breastfeeding yet avoid being viewed as ‘bullies’ by women [87]. A review of models of care provision could inform which models prioritise open communication of women’s goals, and informed and sensitive assessment of the need for individualised care during each woman’s breastfeeding experience. These findings support the evidence that women display greater autonomy about breastfeeding issues when the relationship with the provider enables exploration of options together [88].

This thematic synthesis of published qualitative research has highlighted that experiences in practice and in personal lives can both facilitate and impede breastfeeding support provision. Providers need adequate support to enact their role, and to understand how their positioning shapes their values and contributes to their individual philosophy of breastfeeding support. Context specific experiences may be modifiable through improved prioritisation and resourcing of breastfeeding support services, development of breastfeeding communities of practice within organisations enabling multidisciplinary and multisector learning opportunities, and appropriately implemented and funded relational models of care. Prior personal experiences and challenges in practice that negatively influence support may be reframed through sensitive reflection during formal breastfeeding education and informal debriefing with other support providers in a well-resourced and educated team. Breastfeeding support interventions should be based on evidence of outcome effectiveness [3] women’s perspectives [89, 90] and as this review demonstrates, the context within which the trained provider experiences breastfeeding and breastfeeding support. Continued efforts are needed to enhance experiences which enable effective support, such as learning from experts in practice, being part of an organisation that values breastfeeding, and time to spend with women.

### Implications for practice

The overall contribution of this systematic review of qualitative evidence is a deeper understanding of experiences which influence how breastfeeding support is provided, and the significance of such experiences on the approach taken within support encounters. There are three specific implications for practice. Firstly, development of a multisector breastfeeding team in maternity and family services can harness the enthusiasm and expertise of self-selected healthcare professionals, trained peer supporters and lactation consultants. Shared learning through the integration of regular presentations of case histories to the wider multidisciplinary team will enhance potential for continued development and professional support networks. These teams, working alongside other healthcare professionals should ensure ready access to expertise and referral, and enable other providers to witness confident timely breastfeeding support provision. Secondly, developing and appropriately implementing models of service provision that prioritise relational continuity can enable exposure to longer term routine breastfeeding journeys. Experiencing knowledge of the outcomes of one’s support can build confidence in practice, influencing belief in the value and process of breastfeeding. This will provide the opportunity to build wisdom in the approach to take with certain women, for example when to step in and intervene and when to encourage women to manage their breastfeeding independently underpinned with provider support. Thirdly, consistent full implementation and resourcing of evidence-based breastfeeding support services specifically, and maternity and family services in general, can help organisations ensure that providers are able to enact their role.

### Future research

This systematic review of qualitative evidence provides evidence that the context in which women are supported with breastfeeding in terms of working with colleagues, resourcing and valuing of breastfeeding services, and time in partnership with women has an important and ongoing influence on the development of breastfeeding support knowledge and practice. Due to heterogeneity amongst the included papers, further research with more focus on practice context and the factors which influence practice from the perspective of a range of providers in accredited settings is warranted, for example UNICEF Baby Friendly accreditation which is an international standard of best practice in multi-level breastfeeding support [43]. The potential to optimise the positive factors and minimise the negative factors influencing practice in specific settings may be addressed through evidence-based provider education and service design.

### Strengths & limitations

This review has demonstrated consistency across included papers of common experiences that influence the practice of breastfeeding support providers. Quality appraisal of the studies, data analysis, theme development and application of the CERQual tool have been described in detail in the text and supplementary material, increasing transparency for the reader and trustworthiness of the review. There was high confidence in the descriptive themes *Personal breastfeeding experience*, *Belief in the value and process of breastfeeding* and *Time and resources* due to the adequacy of relevant coherent data and only minor concerns about possible methodological bias.

Methodological limitations within studies include lack of transparency in reporting. No study fulfilled all of the COREQ reporting criteria, with researcher reflexivity under-reported in most studies. Limitations of the review findings in their entirety were assessed using the CERQual tool. Concern about inadequate, irrelevant or incoherent data resulted in the downgrading of some of this review’s descriptive themes from high confidence to moderate or low confidence in findings. There is a paucity of general practitioner, health visitor and trained peer supporter representation in the studies, midwives and lactation consultants predominate in the sample. Some participants identifying as lactation consultants held dual roles, for example as midwife or paediatrician. For the purpose of this study participants were identified as the role-type of the study inclusion criteria in which they participated. Sub-group analysis by role-type was not possible due to heterogeneity of the population. Heterogeneity of methodologies, population demographics and the settings of breastfeeding support provision represented in this review has limited the synthesis and as such the wider transferability of findings. Despite conducting an extensive database search it is possible that not all relevant records were retrieved due to the use of search limiters such as English language and publication date.

## Conclusion

This systematic review has identified personal, professional and workplace experiences which inform the development of a philosophy of breastfeeding support, and experiences which facilitate or hinder expression of that philosophy. This evidence contributes to our understanding of why breastfeeding support interventions can be experienced in a variety of ways by women. The findings suggest the more exposure to effective breastfeeding support that a provider experiences, the richer their philosophy of support becomes. This qualitative evidence synthesis adds to the growing body of literature indicating that successful implementation of complex breastfeeding support interventions requires a deeper understanding of the relational aspects of support between the provider and the woman, and the context within which support is provided.

## Supporting information

S1 Checklist PRISMA 2020 checklist. (DOCX)

S1 Table Table of search histories. (DOCX)

S2 Table Prevalence of themes. (DOCX)

S3 Table CERQual evidence table. (DOCX)

S1 File COREQ appraisal of reporting. (DOCX)

## References

[1] World Health Organization. Global Nutrition Targets 2025: Breastfeeding Policy Brief. 2014 [cited 2021 Feb 9]. Available from: https://www.who.int/publications/i/item/WHO-NMH-NHD-14.7

[2] Victora CG, Bahl R, Barros AJD, França GVA, Horton S, Krasevec J, et al. Breastfeeding in the 21st century: epidemiology, mechanisms, and lifelong effect. The Lancet. 2016;387(10017):475–90. 10.1016/S0140-6736(15)01024-7 26869575

[3] McFadden A, Gavine A, Renfrew MJ, Wade A, Buchanan P, Taylor JL, et al. Support for healthy breastfeeding mothers with healthy term babies. Cochrane Database of Systematic Reviews. 2017;2:CD001141. doi: 10.1002/14651858.CD001141.pub5 28244064 PMC6464485

[4] Horta BL, Loret de Mola C, Victora CG. Long-term consequences of breastfeeding on cholesterol, obesity, systolic blood pressure and type 2 diabetes: a systematic review and meta-analysis. Acta Paediatrica. 2015;104:30–7. doi: 10.1111/apa.13133 26192560

[5] United Nations. Transforming our world: The 2030 Agenda for Sustainable Development.2015 [cited 2021 Feb 9]. Available from: https://www.un.org/sustainabledevelopment/sustainable-development-goals/

[6] Skouteris H, Bailey C, Nagle C, Hauck Y, Bruce L, Morris H. Interventions designed to promote exclusive breastfeeding in high-income countries: a systematic review update. Breastfeeding Medicine. 2017;12(10):604–14. doi: 10.1089/bfm.2017.0065 28885859

[7] Rollins NC, Bhandari N, Hajeebhoy N, Horton S, Lutter CK, Martines JC, et al. Why invest, and what it will take to improve breastfeeding practices? The Lancet. 2016;387(10017):491–504. 10.1016/S0140-6736(15)01044-2 26869576

[8] Thomson G, Trickey H. What works for breastfeeding peer support-time to get real. European Medical Journal: Gynaecology and Obstetrics [Internet]. 2013; (1):[15–22 pp.]. Available from: https://www.emjreviews.com/gynecology-obstetrics/article/what-works-for-breastfeeding-peer-support-time-to-get-real/

[9] Public Health Agency. Health Intelligence Briefing: Breastfeeding in Northern Ireland. 2020.

[10] Renfrew MJ, McCormick FM, Wade A, Quinn B, Dowswell T. Support for healthy breastfeeding mothers with healthy term babies. Cochrane Database of Systematic reviews. 2012(5):CD001141. doi: 10.1002/14651858.CD001141.pub4 22592675 PMC3966266

[11] McInnes RJ, Chambers JA. Supporting breastfeeding mothers: qualitative synthesis. Journal of Advanced Nursing. 2008;62(4):407–27. doi: 10.1111/j.1365-2648.2008.04618.x 18476941

[12] Blixt I, Johansson M, Hildingsson I, Papoutsi Z, Rubertsson C. Women’s advice to healthcare professionals regarding breastfeeding: "offer sensitive individualized breastfeeding support"—An interview study. International Breastfeeding Journal. 2019;14(1). doi: 10.1186/s13006-019-0247-4 31889974 PMC6916109

[13] McAndrew F, Thompson J, Fellows L, Large A, Speed M, Renfrew MJ. Infant feeding survey 2010 Leeds: Health and Social Care Information Centre; 2012. Available from: http://doc.ukdataservice.ac.uk/doc/7281/mrdoc/pdf/7281_ifs-uk-2010_report.pdf.

[14] Tarrant M, Dodgson JE, Wu KM. Factors contributing to early breast-feeding cessation among Chinese mothers: an exploratory study. Midwifery. 2014;30(10):1088–95. doi: 10.1016/j.midw.2014.03.002 24703632

[15] Oakley LL, Henderson J, Redshaw M, Quigley MA. The role of support and other factors in early breastfeeding cessation: an analysis of data from a maternity survey in England. BMC pregnancy and childbirth. 2014;14(1):88. 24571566 10.1186/1471-2393-14-88 PMC3939405

[16] Schmied V, Beake S, Sheehan A, McCourt C, Dykes F. Women’s perceptions and experiences of breastfeeding support: a metasynthesis. Birth. 2011;38(1):49–60. doi: 10.1111/j.1523-536X.2010.00446.x 21332775

[17] Taylor AM, Teijlingen Ev, Ryan KM, Alexander J. ’Scrutinised, judged and sabotaged’: A qualitative video diary study of first-time breastfeeding mothers. Midwifery. 2019;75:16–23. doi: 10.1016/j.midw.2019.04.004 30981161

[18] Leeming D, Williamson I, Johnson S, Lyttle S. Making use of expertise: A qualitative analysis of the experience of breastfeeding support for first-time mothers. Maternal and Child Nutrition. 2015;11(4):687–702. doi: 10.1111/mcn.12033 23557351 PMC6860267

[19] Marks D, O’Connor R. Health professionals’ attitudes towards the promotion of breastfeeding. British Journal of Midwifery. 2015;23(1):50–8.

[20] Moore GF, Audrey S, Barker M, Bond L, Bonell C, Hardeman W, et al. Process evaluation of complex interventions: Medical Research Council guidance. British Medical Journal [Internet]. 2015 [cited 2021 Feb 13]; 350:[1258 p.]. doi: 10.1136/bmj.h1258 25791983 PMC4366184

[21] Jolly K, Ingram L, Khan KS, Deeks JJ, Freemantle N, MacArthur C. Systematic review of peer support for breastfeeding continuation: metaregression analysis of the effect of setting, intensity, and timing. BMJ [Internet]. 2012 [cited 2021 Feb 13]; 344. doi: 10.1136/bmj.d8287 22277543

[22] Chetwynd EM, Wasser HM, Poole C. Breastfeeding Support Interventions by International Board Certified Lactation Consultants: A Systemic Review and Meta-Analysis. Journal of Human Lactation [Internet]. 2019 [cited 2021 Mar 10]; 35(3). doi: 10.1177/0890334419851482 31206317

[23] Patnode CD, Henninger ML, Senger CA, Perdue LA, Whitlock EP. Primary care interventions to support breastfeeding: updated evidence report and systematic review for the US Preventive Services Task Force. JAMA. 2016;316(16):1694–705. doi: 10.1001/jama.2016.8882 27784101 PMC13475363

[24] Swerts M, Westhof E, Bogaerts A, Lemiengre J. Supporting breast-feeding women from the perspective of the midwife: A systematic review of the literature. Midwifery. 2016;37:32–40. doi: 10.1016/j.midw.2016.02.016 27217235

[25] Page M, McKenzie J, Bossuyt P, Boutron I, Hoffmann T, Mulrow C, et al. The PRISMA 2020 statement: an updated guideline for reporting systematic reviews. BMJ. 2021;372. 10.1136/bmj.n71 PMC8005924 33782057

[26] Chesnel M, Healy M, McNeill J. The experiences of trained breastfeeding support providers that influence how breastfeeding support is practised: A protocol for a qualitative evidence synthesis. PLOS One. 2021;16(7):e0254445. doi: 10.1371/journal.pone.0254445 34324535 PMC8321120

[27] Khan K, Kunz R, Kleijnen J, Antenes G. Systematic reviews to support evidence-based medicine. 2nd ed. London: Hodder and Staughton; 2011.

[28] The World Bank. World Bank Country and Lending Groups 2020. Available from: https://datahelpdesk.worldbank.org/knowledgebase/articles/906519-world-bank-country-and-lending-groups.

[29] Nursing and Midwifery Council. Standards of proficiency for midwives. London: Nursing & Midwifery Council; 2019.

[30] World Health Organization. Global strategy for infant and young child feeding.2003 [cited 2021 Mar 10]. Available from: https://apps.who.int/iris/bitstream/handle/10665/42590/9241562218.pdf?sequence=1

[31] Healy M, Nyman V, Spence D, Otten R, Verhoeven C. How do midwives facilitate women to give birth during physiological second stage of labour? A systematic review. PLOS ONE. 2020;15(7):e0226502. doi: 10.1371/journal.pone.0226502 32722680 PMC7386622

[32] Critical Appraisal Skills Programme. CASP Qualitative Checklist. 2018 [cited 2021 18th August]. Available from: www.casp-uk.net.

[33] Tong A, Sainsbury P, Craig J. Consolidated criteria for reporting qualitative research (COREQ): a 32-item checklist for interviews and focus groups. International Journal for Quality in Health Care. 2007;19(6):349–57. doi: 10.1093/intqhc/mzm042 17872937

[34] Lewin S, Booth A, Glenton C, Munthe-Kaas H, Rashidian A, Wainwright M, et al. Applying GRADE-CERQual to qualitative evidence synthesis findings: introduction to the series. Implementation Science [Internet]. 2018 [cited 2021 Feb 16]. doi: 10.1186/s13012-017-0688-3 29384079 PMC5791040

[35] Thomas J, Harden A. Methods for the thematic synthesis of qualitative research in systematic reviews. BMC Medical Research Methodology [Internet]. 2008 [cited 2021 Mar 11]; 8(1). doi: 10.1186/1471-2288-8-45 18616818 PMC2478656

[36] Braun V, Clarke V. Using thematic analysis in psychology. Qualitative Research in Psychology. 2006;3(2):77–101.

[37] Braun V, Clarke V. What can “thematic analysis” offer health and wellbeing researchers? International journal of qualitative studies on health and well-being. 2014;9. doi: 10.3402/qhw.v9.26152 25326092 PMC4201665

[38] Braun V, Clarke V. One size fits all? What counts as quality practice in (reflexive) thematic analysis? Qualitative Research in Psychology. 2020:1–25.

[39] Lucchini-Raies C, Marquez-Doren F, Garay Unjidos N, Contreras Veliz J, Jara Suazo D, Calabacero Florechaes C, et al. Care during Breastfeeding: Perceptions of Mothers and Health Professionals. Investigacion y Educacion en Enfermeria. 2019;37(2). doi: 10.17533/udea.iee.v37n2e09 31487446 PMC7871484

[40] Hopper H, Skirton H. Factors influencing the sustainability of volunteer peer support for breast-feeding mothers within a hospital environment: An exploratory qualitative study. Midwifery. 2016;32:58–65. doi: 10.1016/j.midw.2015.09.007 26453506

[41] Torres JM. Medicalizing to demedicalize: Lactation consultants and the (de) medicalization of breastfeeding. Social Science & Medicine. 2014;100:159–66. doi: 10.1016/j.socscimed.2013.11.013 24444851

[42] Burns E, Schmied V. "The right help at the right time": Positive constructions of peer and professional support for breastfeeding. Women Birth. 2017;30(5):389–97. doi: 10.1016/j.wombi.2017.03.002 28359753

[43] UNICEF Baby Friendly Initiative. A Guide to the Unicef UK Baby Friendly Initiative Standards 2017. Available from: https://www.unicef.org.uk/babyfriendly/wp-content/uploads/sites/2/2014/02/Guide-to-the-Unicef-UK-Baby-Friendly-Initiative-Standards.pdf.

[44] Swerts M, Westhof E, Lemiengre J, Bogaerts A. The supporting role of the midwife during the first 14 days of breastfeeding: A descriptive qualitative study in maternity wards and primary healthcare. Midwifery. 2019;78:50–7. doi: 10.1016/j.midw.2019.07.016 31357116

[45] Edwards ME, Jepson RG, McInnes RJ. Breastfeeding initiation: An in-depth qualitative analysis of the perspectives of women and midwives using Social Cognitive Theory. Midwifery. 2018;57:8–17. doi: 10.1016/j.midw.2017.10.013 29175258

[46] Furber CM, Thomson AM. Midwives in the UK: an exploratory study of providing newborn feeding support for postpartum mothers in the hospital. Journal of Midwifery & Women’s Health. 2007;52(2):142–7. doi: 10.1016/j.jmwh.2006.10.016 17336820

[47] Furber CM, Thomson AM. The emotions of integrating breastfeeding knowledge into practice for English midwives: a qualitative study. International Journal of Nursing Studies. 2008a;45(2):286–97. doi: 10.1016/j.ijnurstu.2006.08.017 17049354

[48] Furber CM, Thomson AM. Breastfeeding practice in the UK: midwives’ perspectives. Maternal and Child Nutrition. 2008b;4(1):44–54. doi: 10.1111/j.1740-8709.2007.00094.x 18171406 PMC6860633

[49] Lewin S, Bohren M, Rashidian A, Munthe-Kaas H, Glenton C, Colvin CJ, et al. Applying GRADE-CERQual to qualitative evidence synthesis findings—paper 2: how to make an overall CERQual assessment of confidence and create a Summary of Qualitative Findings table. Implementation Science [Internet]. 2018 [cited 2021 Mar 13]; 13(1). doi: 10.1186/s13012-017-0689-2 29384082 PMC5791047

[50] Munthe-Kaas H, Bohren MA, Glenton C, Lewin S, Noyes J, Tunçalp Ö, et al. Applying GRADE-CERQual to qualitative evidence synthesis findings—paper 3: how to assess methodological limitations. Implementation Science [Internet]. 2018 [cited 2021 Mar 13]; 13(1). doi: 10.1186/s13012-017-0690-9 29384078 PMC5791044

[51] Colvin CJ, Garside R, Wainwright M, Munthe-Kaas H, Glenton C, Bohren MA, et al. Applying GRADE-CERQual to qualitative evidence synthesis findings—paper 4: how to assess coherence. Implementation Science. 2018;13(1):13. doi: 10.1186/s13012-017-0691-8 29384081 PMC5791039

[52] Glenton C, Carlsen B, Lewin S, Munthe-Kaas H, Colvin CJ, Tunçalp Ö, et al. Applying GRADE-CERQual to qualitative evidence synthesis findings—paper 5: how to assess adequacy of data. Implementation Science. 2018;13(1):14. doi: 10.1186/s13012-017-0692-7 29384077 PMC5791045

[53] Noyes J, Booth A, Lewin S, Carlsen B, Glenton C, Colvin CJ, et al. Applying GRADE-CERQual to qualitative evidence synthesis findings–paper 6: how to assess relevance of the data. Implementation Science [Internet]. 2018 [cited 2021 Mar 12]; 13(1). doi: 10.1186/s13012-017-0693-6 29384080 PMC5791042

[54] Anstey EH, Coulter M, Jevitt CM, Perrin KM, Dabrow S, Klasko-Foster LB, et al. Lactation Consultants’ Perceived Barriers to Providing Professional Breastfeeding Support. Journal of Human Lactation. 2018;34(1):51–67. doi: 10.1177/0890334417726305 28820951

[55] Dunne S, Fallon A. Public health nurses’ experiences of supporting women to breastfeed in community settings in Ireland. Journal of Health Visiting. 2020;8(6):240–6.

[56] Lawton K, Robinson A. Midwives’ experiences of helping women struggling to breastfeed. British Journal of Midwifery. 2016;24(4):248–53.

[57] Marshall JL, Renfrew MJ, Godfrey M. Using evidence in practice: what do health professionals really do? A study of care and support for breastfeeding women in primary care. Clinical Effectiveness in Nursing. 2006;9:e181–e90.

[58] Nelson AM. Maternal-newborn nurses’ experiences of inconsistent professional breastfeeding support. Journal of Advanced Nursing. 2007;60(1):29–38. doi: 10.1111/j.1365-2648.2007.04373.x 17824937

[59] Tennant R, Wallace LM, Law S. Barriers to breastfeeding: a qualitative study of the views of health professionals and lay counsellors. Community Practitioner. 2006;79(5):152–6. 16724480

[60] Wright AI, Hurst NM. Personal Infant Feeding Experiences of Postpartum Nurses Affect How They Provide Breastfeeding Support. JOGNN—Journal of Obstetric, Gynecologic, and Neonatal Nursing. 2018;47(3):342–51. doi: 10.1016/j.jogn.2018.02.011 29604239

[61] Burns E, Fenwick J, Sheehan A, Schmied V. Mining for liquid gold: midwifery language and practices associated with early breastfeeding support. Maternal and Child Nutrition. 2013;9(1):57–73. doi: 10.1111/j.1740-8709.2011.00397.x 22405753 PMC6860896

[62] Dykes F. A critical ethnographic study of encounters between midwives and breast-feeding women in postnatal wards in England. Midwifery. 2005;21(3):241–52. doi: 10.1016/j.midw.2004.12.006 15967551

[63] Burns E, Schmied V, Fenwick J, et al. Liquid gold from the milk bar: Constructions of breastmilk and breastfeeding women in the language and practices of midwives. Social Science and Medicine. 2012;75(10):1737–45. doi: 10.1016/j.socscimed.2012.07.035 22906527

[64] Bäckström CA, Wahn EIH, Ekström AC. Two sides of breastfeeding support: experiences of women and midwives. International Breastfeeding Journal. 2010;5:8p-p. 21114812 10.1186/1746-4358-5-20 PMC3001698

[65] Noel-Weiss J, Cragg B, Woodend AK. Exploring how IBCLCs manage ethical dilemmas: a qualitative study. BMC Medical Ethics. 2012;13:8. 22824376 10.1186/1472-6939-13-18 PMC3467169

[66] Renfrew MJ. Time to get serious about educating health professionals. Maternal & child nutrition. 2006;2(4):193–5. doi: 10.1111/j.1740-8709.2006.00075.x 16999764 PMC6860784

[67] Dykes F. The education of health practitioners supporting breastfeeding women: time for critical reflection. Maternal & child nutrition. 2006;2(4):204–16. doi: 10.1111/j.1740-8709.2006.00071.x 16999766 PMC6860707

[68] Brown A, Chucha S, Trickey H. Becoming breastfeeding friendly in Wales: Recommendations for scaling up breastfeeding support. Maternal & Child Nutrition. 2022:e13355. doi: 10.1111/mcn.13355 35403354 PMC9835566

[69] Pérez‐Escamilla R, Hromi‐Fiedler AJ, Gubert MB, Doucet K, Meyers S, dos Santos Buccini G. Becoming Breastfeeding Friendly Index: Development and application for scaling‐up breastfeeding programmes globally. Maternal & Child Nutrition. 2018;14(3):e12596. doi: 10.1111/mcn.12596 29468832 PMC6866061

[70] Abbott S, Renfrew MJ, McFadden A. ‘Informal’learning to support breastfeeding: local problems and opportunities. Maternal & Child Nutrition. 2006;2(4):232–8. doi: 10.1111/j.1740-8709.2006.00069.x 16999768 PMC6860644

[71] Prokop N, Meedya S, Sim J. Integrative Review of the Experiences of Registered Nurses Who Support Breastfeeding Women. Journal of obstetric, gynecologic, and neonatal nursing: JOGNN. 2021;50(3):266–74. doi: 10.1016/j.jogn.2021.02.003 33689688

[72] Reddin E, Pincombe J, Darbyshire P. Passive resistance: early experiences of midwifery students/graduates and the Baby Friendly Health Initiative 10 steps to successful breastfeeding. Women & Birth: Journal of the Australian College of Midwives. 2007;20(2):71–6. doi: 10.1016/j.wombi.2007.04.003 17493886

[73] Garner CD, Ratcliff SL, Thornburg LL, Wethington E, Howard C, Rasmussen K. Discontinuity of Breastfeeding Care: "There’s No Captain of the Ship". Breastfeeding Medicine. 2016;11(1):32–9. doi: 10.1089/bfm.2015.0142 26566010 PMC4742991

[74] Chang Y-S, Beake MS, Kam MJ, Lok KY-W, Bick D. Views and experiences of women, peer supporters and healthcare professionals on breastfeeding peer support: A systematic review of qualitative studies. Midwifery. 2022:103299. doi: 10.1016/j.midw.2022.103299 35276491

[75] Dykes F. Breastfeeding in hospital: Mothers, midwives and the production line.2006.

[76] McLelland G, Hall H, Gilmour C, Cant R. Support needs of breast-feeding women: views of Australian midwives and health nurses. Midwifery. 2015;31(1):e1–6. doi: 10.1016/j.midw.2014.09.008 25451547

[77] Gleeson D, Flowers K, Fenwick J. Midwives’ Time and Presence: A Key Factor in Facilitating Breastfeeding Support for New Mothers. International Journal of Childbirth. 2014;4(4):219–27.

[78] Perez-Escamilla R, Chapman DJ. Breastfeeding protection, promotion, and support in the United States: a time to nudge, a time to measure. J Hum Lact. 2012;28(2):118–21. doi: 10.1177/0890334412436721 22526338

[79] Ranch MM, Jämtén S, Thorstensson S, Ekström-Bergström AC. First-time mothers have a desire to be offered professional breastfeeding support by pediatric nurses: an evaluation of the mother-perceived-professional support scale. Nursing Research and Practice [Internet]. 2019 [cited 2021 Feb 13]. doi: 10.1155/2019/8731705 31467709 PMC6699260

[80] Redshaw M, Henderson J. Learning the hard way: expectations and experiences of infant feeding support. Birth. 2012;39(1):21–9. doi: 10.1111/j.1523-536X.2011.00509.x 22369602

[81] Grant A, McEwan K, Tedstone S, Greene G, Copeland L, Hunter B, et al. Availability of breastfeeding peer support in the United Kingdom: A cross-sectional study. Maternal and Child Nutrition. 2018;14(1). doi: 10.1111/mcn.12476 28685958 PMC6865891

[82] Stockdale DJ, Sinclair M, Kernohan W, Keller J. Understanding motivational theory and the psychology of breastfeeding. Theory for midwifery practice. 2nd ed. ed: Palgrave Macmillan; 2011. p. 92–106.

[83] Prussing E, Browne G, Dowse E, Wilson A. Implementing midwifery continuity of care models in NSW regional areas. Women and Birth. 2018;31:S5–S6. 10.1016/j.wombi.2022.03.006 35410848

[84] Homer CS, Leap N, Edwards N, Sandall J. Midwifery continuity of carer in an area of high socio-economic disadvantage in London: a retrospective analysis of Albany Midwifery Practice outcomes using routine data (1997–2009). Midwifery. 2017;48:1–10. doi: 10.1016/j.midw.2017.02.009 28284877

[85] Alianmoghaddam N, Phibbs S, Benn C. Resistance to breastfeeding: A Foucauldian analysis of breastfeeding support from health professionals. Women & Birth: Journal of the Australian College of Midwives [Internet]. 2017 [cited 2021 Feb 13]; 30(6):[e281-e91 pp.]. doi: 10.1016/j.wombi.2017.05.005 28648583

[86] Gustafsson I, Nystrom M, Palmer L. Midwives’ lived experience of caring for new mothers with initial breastfeeding difficulties: A phenomenological study. Sexual & reproductive healthcare: official journal of the Swedish Association of Midwives. 2017;12:9–15. doi: 10.1016/j.srhc.2016.12.003 28477939

[87] Walsh A, Pincombe J, Henderson A. An Examination of Maternity Staff Attitudes Towards Implementing Baby Friendly Health Initiative (BFHI) Accreditation in Australia. Maternal & Child Health Journal. 2011;15(5):597–609. doi: 10.1007/s10995-010-0628-1 20571902

[88] Quinn EM, Gallagher L, de Vries J. A qualitative exploration of breastfeeding support groups in Ireland from the women’s perspectives. Midwifery. 2019;78:71–7. doi: 10.1016/j.midw.2019.08.001 31398596

[89] Brown A. What do women really want? Lessons for breastfeeding promotion and education. Breastfeeding medicine. 2016;11(3):102–10. doi: 10.1089/bfm.2015.0175 27052189

[90] Graffy J, Taylor J. What information, advice, and support do women want with breastfeeding? Birth. 2005;32(3):179–86. doi: 10.1111/j.0730-7659.2005.00367.x 16128971

