# Supplementary material for: Experiences that influence how trained providers support women with breastfeeding: A systematic review of qualitative evidence
Source: PLoS One. 2022 Oct 14;17(10):e0275608. doi: 10.1371/journal.pone.0275608 (PMC9565393; doi:10.1371/journal.pone.0275608)
Supplement: S1 Table — (DOCX) [file pone.0275608.s002.docx]

S1: Table of Search histories

Medline:

| Population:  **Trained breastfeeding support providers** | Exposure:  **Breastfeeding and breastfeeding support provision** | Context:  **Experiences that influence breastfeeding support practices** | Type of study:  **Studies with qualitative methods and findings** |
| --- | --- | --- | --- |
| Limited to English language and year of publication 2003 - current | | | |
| Midwifery/OR Nurse midwives/OR Midwi*.mp  OR Health Personnel/ OR Physicians/ OR Doctors.mp  OR Health visitors or nurses, community health/  OR Peer counselling.mp OR Volunteers/  OR Lactation Consultants.mp OR Breastfeeding counsellors.mp OR Breastfeeding supporters.mp OR Doulas.mp or Doulas/ | Breastfeeding promotion.mp  OR Breastfeeding support.mp  OR Breastfeeding.mp or Breastfeeding/  OR Lactation management.mp  OR Infant Feeding.mp  OR Breastfeeding Counseling | Experiences.mp perception*.mp  OR views.mp  OR Feelings.mp or Emotions/ | Qualitative Research/ or qualitative.mp  OR Interviews.mp  OR Focus groups.mp OR “mixed method*”  OR Ethnography  OR Participant observation |
| Four PEOT search terms combined with **AND** | | | |

Web of Science:

| Population:  **Trained breastfeeding support providers** | Exposure:  **Breastfeeding support provision** | Context:  **Experiences that influence breastfeeding support practices** | Type of study:  **Studies with qualitative methods and findings** |
| --- | --- | --- | --- |
| Limited to English language and year of publication 2003 - current | | | |
| ("breastfeeding supporter*") OR (Doula*) OR (midwi*) OR ("nurse midw*") OR ("health personnel") OR ("healthcare staff") OR (physician*) OR (doctor*) OR ("health visitor*") OR ("peer support*") OR ("Peer counselling") OR (volunteer*) OR ("lactation consultant*") OR ("breastfeeding counsellor*") | ("breastfeeding support") OR ("Breastfeeding promotion") OR m(Breastfeeding) OR ("lactation management") OR ("Infant feeding") OR (breastfeeding counsel*) | (experience*) OR (perception*) OR (views) OR (opinion*) OR (attitude*) OR (Emotion*) OR (Feeling*) OR ("attitude* to breastfeeding") | ("mixed method*") OR ("focus groups") OR ("interviews") OR ("qualitative analysis") OR ("qualitative research") OR ("ethnography") OR ("participant observation") |
| Four PEOT search terms combined with **AND** | | | |

Scopus:

| Population:  **Trained breastfeeding support providers** | Exposure:  **Breastfeeding support provision** | Context:  **Experiences that influence breastfeeding support practices** | Type of study:  **Studies with qualitative methods and findings** |
| --- | --- | --- | --- |
| Limited to English language and year of publication 2003 - current | | | |
| ( midwi* ) OR ( "nurse midwi*" ) OR ( "health personnel" ) OR ( "healthcare staff" ) OR ( physician* ) OR ( doctor* ) OR ( "health visitor*" ) OR ( "peer support*" ) OR ( "peer counselling" ) ( volunteer* ) OR ( "lactation consultant*" ) OR ( "breastfeeding counsellor*" ) OR ( "breastfeeding supporter*" ) OR ( doula* ) | ( "breastfeeding support" ) OR ( "breastfeeding promotion" ) OR ( "breastfeeding education" ) OR ( breastfeeding ) OR ( "lactation management" ) OR ( "infant feeding" ) OR ( breastfeeding AND counsel* ) | ( experienc* ) OR ( perception* ) OR ( views ) OR  ( emotion* ) OR ( feeling* ) | ( "qualitative research" ) OR ( "qualitative analysis" ) OR ( interview* ) OR ( "group interview*" ) OR ( "focus group*" ) OR ( "ethnography" ) OR ( "participant observation" ) |
| Four PEOT search terms combined with **AND** | | | |

APA PsycInfo (Ovid):

| Population:  **Trained breastfeeding support providers** | Exposure:  **Breastfeeding support provision** | Context:  **Experiences that influence breastfeeding support practices** | Type of study:  **Studies with qualitative methods and findings** |
| --- | --- | --- | --- |
| Limited to English language and year of publication 2003 - current | | | |
| peer support*.mp  lactation consultants.mp.  breastfeeding counsellors.mp.  breastfeeding supporters.mp.  doulas.mp. or Doulas/  Midwifery.mp. or exp Midwifery/  Midwi*.mp.  exp Health Personnel/  doctors.mp. or exp Physicians/  exp Peer Counseling/  volunteers.mp. or exp Volunteers/  health visitor.mp.  nurse midwives.mp.  health visitor*.mp. | Breastfeeding promotion.mp.  breastfeeding support.mp.  Breastfeeding.mp. or Breast Feeding/  lactation management.mp.  infant feeding.mp.  perception*.mp.  breastfeeding counsel*.mp.  breastfeeding education.mp. | Experiences.mp. or exp "Experiences (Events)"/  Views.mp.  Feelings.mp. or exp Emotions/ | Qualitative Research/ or qualitative.mp  interviews.mp.  focus groups.mp.  "mixed method*".mp  exp Ethnography/  exp Participant Observation/ |
| Four PEOT search terms combined with **AND** | | | |

CINAHL + :

| Population:  **Trained breastfeeding support providers** | Exposure:  **Breastfeeding support provision** | Context:  **Experiences that influence breastfeeding support practices** | Type of study:  **Studies with qualitative methods and findings** |
| --- | --- | --- | --- |
| Limited to English language and year of publication 2003 - current | | | |
| (MH "Midwives") OR (MH "Nurse Midwives")  (MH "Midwifery") OR (MH "Nurse Midwifery")  (MH "Peer Counseling")  "breastfeeding counsellor"  "breastfeeding supporter"  "peer support*"  (MH "Community Health Nursing") OR "health visitors"  (MH "Doulas") OR "doulas"  (MH "Lactation Consultants") OR "lactation consultant"  "volunteer*"  (MH "Health Personnel") | (MH "Infant Feeding") OR "infant feeding"  "lactation management"  "breastfeeding support"  (MH "Breast Feeding") OR (MH "Breast Feeding Promotion")  "Breastfeeding counsel*" | (MH "Emotions") OR "feelings"  (MH "Life Experiences") OR (MH "Work Experiences") OR (MH "Volunteer Experiences") OR "experiences"  "views"  (MH "Perception") OR "perceptions" | (MH "Participant Observation")  "ethnography"  ""mixed method*""  "focus groups"  "interviews"  (MH "Qualitative Studies") OR "qualitative" |
| Four PEOT search terms combined with **AND** | | | |

Embase :

| Population:  **Trained breastfeeding support providers** | Exposure:  **Breastfeeding support provision** | Context:  **Experiences that influence breastfeeding support practices** | Type of study:  **Studies with qualitative methods and findings** |
| --- | --- | --- | --- |
| Limited to English language and year of publication 2003 - current | | | |
| health care personnel/  midwives.mp. or midwife/  nurse midwife/  nurse midwifery/  midwi*.mp.  health visitor.mp. or health visitor/  doctors.mp. or physician/  peer support*.mp.  volunteer/  peer counseling/  lactation consultants.mp. or lactation consultant/  doulas.mp. or doula/  breastfeeding counsellors.mp.  breastfeeding supporters.mp. | breast feeding/ or breastfeeding.mp.  breastfeeding support.mp. or breast feeding education/  breastfeeding promotion.mp.  lactation management.mp.  infant feeding/    breastfeeding counsel*.mp | experience/  perception/  "Attitudes to breastfeeding".mp.  attitude/  emotion/  feelings.mp.  Opinions.mp.  views.mp. | qualitative research/ or qualitative analysis/ or Qualitative.mp.  interviews.mp.  focus groups.mp.  "mixed method*".mp.  participant observation/  ethnography/ |
| Four PEOT search terms combined with **AND** | | | |

Maternity and Infant Care

| Population:  **Trained breastfeeding support providers** | Exposure:  **Breastfeeding support provision** | Context:  **Experiences that influence breastfeeding support practices** | Type of study:  **Studies with qualitative methods and findings** |
| --- | --- | --- | --- |
| Limited to English language and year of publication 2003 - current | | | |
| Midwives.de. or midwives.mp.  Nurse-midwives.de.  health personnel.mp. or Health personnel.de.  Physicians.de. or physicians.mp.  doctors.mp. or Doctors.de.  Health visitors.de. or health visitors.mp.  peer support*.mp. or Peer support.de.  Volunteers.de. or volunteer.mp.  peer counselling.mp.  Lactation consultants.de. or lactation consultants.mp.  midwifery.mp. or Midwifery.de.  Doulas.de. or doulas.mp. | breastfeeding.mp. or Breastfeeding.de.  breastfeeding support.mp.  breastfeeding education.mp. or Breastfeeding - education.de.  Breastfeeding promotion.de. or Breastfeeding promotion.mp.  Infant Feeding.mp. or Infant feeding.de.  lactation management.mp  breastfeeding counsel*.mp. | experiences.mp. or Experiences.de.  Perceptions.de. or Perceptions.mp.  Opinions.mp.  views.mp  "Views (midwives)".de.  Emotions.de. | qualitative.mp. or Qualitative research.de.    interviews.mp.  focus groups.mp  "mixed method*".mp  ethnography.mp.  participant observation.mp |
| Four PEOT search terms combined with **AND** | | | |
