# Supplementary material for: Experiences that influence how trained providers support women with breastfeeding: A systematic review of qualitative evidence
Source: PLoS One. 2022 Oct 14;17(10):e0275608. doi: 10.1371/journal.pone.0275608 (PMC9565393; doi:10.1371/journal.pone.0275608)
Supplement: S2 Table — (DOCX) [file pone.0275608.s003.docx]

S2: Prevalence of themes

| DESCRIPTIVE THEMES |  |  | PAPER REFERENCES | | | | | | | | | | | | | | | | | | |
| --- | --- | --- | --- | --- | --- | --- | --- | --- | --- | --- | --- | --- | --- | --- | --- | --- | --- | --- | --- | --- | --- |
|  | 1 | 2 | 3 | 4 | 5 | 6 | 7 | 8 | 9 | 10 | 11 | 12 | 13 | 14 | 15 | 16 | 17 | 18 | 19 | 20 | 21 |
| Personal breastfeeding experience | ● |  |  |  | ● | ● |  | ● |  |  |  | ● | ● | ● | ● | ● |  | ● | ● |  | ● |
| Belief in the value and process of breastfeeding |  |  | ● | ● | ● |  | ● | ● |  |  |  | ● |  | ● |  | ● |  |  |  | ● | ● |
| knowledge FoR practice |  | ● | ● | ● |  |  | ● | ● |  | ● | ● | ● | ● | ● | ● |  |  | ● | ● |  | ● |
| Collaboration | ● | ● |  |  |  | ● |  |  |  |  | ● |  |  |  |  |  |  |  |  |  |  |
| Inconsistency in support and advice | ● | ● |  |  |  | ● |  | ● |  |  | ● |  |  |  |  | ● |  |  |  |  |  |
| Opinions of others |  |  |  | ● |  | ● |  | ● |  | ● |  |  | ● |  |  |  |  | ● |  |  |  |
| Time and resources | ● |  |  | ● | ● | ● | ● | ● | ● | ● | ● | ● | ● |  |  | ● |  | ● |  |  |  |
| Organisational values | ● |  |  |  |  | ● |  | ● |  | ● |  |  |  |  |  |  |  |  |  |  |  |
| Expectation of role |  |  | ● |  |  |  |  | ● | ● | ● |  |  | ● |  |  | ● |  |  |  | ● |  |
| Perceptions of mothers breastfeeding reality | ● | ● | ● | ● | ● | ● | ● | ● | ● | ● | ● |  | ● |  |  | ● |  | ● |  | ● |  |
| Relationship and communication | ● | ● | ● | ● | ● |  | ● |  |  | ● | ● |  | ● | ● | ● | ● | ● | ● |  | ● | ● |

Mapping of analytical themes

| ANALyTICAL THEMES |  |  | PAPER REFERENCES | | | | | | | | | | | | | | | | | | |
| --- | --- | --- | --- | --- | --- | --- | --- | --- | --- | --- | --- | --- | --- | --- | --- | --- | --- | --- | --- | --- | --- |
|  | 1 | 2 | 3 | 4 | 5 | 6 | 7 | 8 | 9 | 10 | 11 | 12 | 13 | 14 | 15 | 16 | 17 | 18 | 19 | 20 | 21 |
| A PERSONAL Philosophy of breastfeeding support | ● | ● | ● | ● | ● | ● | ● | ● |  | ● | ● | ● | ● | ● | ● | ● | ● | ● | ● | ● | ● |
| Encounters with BREASTFEEDING WOMEN | ● | ● | ● | ● | ● | ● | ● | ● | ● | ● | ● | ● | ● | ● | ● | ● | ● | ● |  | ● | ● |
| Collegiate relations/teamwork | ● | ● |  | ● |  | ● | ● | ● |  | ● | ● |  | ● |  |  | ● | ● | ● | ● | ● |  |
| NEGOTIATING Organisational constraints | ● |  | ● |  | ● | ● | ● | ● | ● | ● | ● | ● | ● |  |  | ● |  | ● |  | ● |  |

References

1. Anstey EH, Coulter M, Jevitt CM, Perrin KM, Dabrow S, Klasko-Foster LB, et al. Lactation consultants' perceived barriers to providing professional breastfeeding support. Journal of Human Lactation. 2018;34(1):51-67.

2. Backstrom CA, Wahn E, Ekstrom AC. Two sides of breastfeeding support: experiences of women and midwives. International Breastfeeding Journal. 2010;5(20):8.

3. Burns E, Schmied V, Fenwick J, et al. Liquid gold from the milk bar: Constructions of breastmilk and breastfeeding women in the language and practices of midwives. Social Science and Medicine. 2012;75(10):1737-45.

4. Burns E, Fenwick J, Sheehan A, Schmied V. Mining for liquid gold: midwifery language and practices associated with early breastfeeding support. Maternal and Child Nutrition. 2013;9(1):57-73.

5. Burns E, Schmied V. "The right help at the right time": Positive constructions of peer and professional support for breastfeeding. Women Birth. 2017;30(5):389-97.

6. Dunne S, Fallon A. Public health nurses' experiences of supporting women to breastfeed in community settings in Ireland. Journal of Health Visiting. 2020;8(6):240-6.

7. Dykes F. A critical ethnographic study of encounters between midwives and breast-feeding women in postnatal wards in England. Midwifery. 2005;21(3):241-52.

8. Edwards ME, Jepson RG, McInnes RJ. Breastfeeding initiation: An in-depth qualitative analysis of the perspectives of women and midwives using Social Cognitive Theory. Midwifery. 2018;57:8-17.

9. Furber CM, Thomson AM. Midwives in the UK: an exploratory study of providing newborn feeding support for postpartum mothers in the hospital. Journal of Midwifery & Women's Health. 2007;52(2):142-7.

10. Furber CM, Thomson AM. The emotions of integrating breastfeeding knowledge into practice for English midwives: a qualitative study. International Journal of Nursing Studies. 2008a;45(2):286-97.

11. Furber CM, Thomson AM. Breastfeeding practice in the UK: midwives' perspectives. Maternal and Child Nutrition. 2008b;4(1):44-54.

12. Hopper H, Skirton H. Factors influencing the sustainability of volunteer peer support for breast-feeding mothers within a hospital environment: An exploratory qualitative study. Midwifery. 2016;32:58-65.

13. Lawton K, Robinson A. Midwives' experiences of helping women struggling to breastfeed. British Journal of Midwifery. 2016;24(4):248-53.

14. Lucchini-Raies C, Marquez-Doren F, Garay Unjidos N, Contreras Veliz J, Jara Suazo D, Calabacero Florechaes C, et al. Care during Breastfeeding: Perceptions of Mothers and Health Professionals. Investigacion y Educacion en Enfermeria. 2019;37(2).

15. Marshall JL, Renfrew MJ, Godfrey M. Using evidence in practice: what do health professionals really do? A study of care and support for breastfeeding women in primary care. Clinical Effectiveness in Nursing. 2006;9:e181-e90.

16. Nelson AM. Maternal-newborn nurses' experiences of inconsistent professional breastfeeding support. Journal of Advanced Nursing. 2007;60(1):29-38.

17. Noel-Weiss J, Cragg B, Woodend AK. Exploring how IBCLCs manage ethical dilemmas: a qualitative study. BMC Medical Ethics. 2012;13:8.

18. Swerts M, Westhof E, Lemiengre J, Bogaerts A. The supporting role of the midwife during the first 14 days of breastfeeding: A descriptive qualitative study in maternity wards and primary healthcare. Midwifery. 2019;78:50-7.

19. Tennant R, Wallace LM, Law S. Barriers to breastfeeding: a qualitative study of the views of health professionals and lay counsellors. Community Practitioner. 2006;79(5):152-6.

20. Torres JM. Medicalizing to demedicalize: Lactation consultants and the (de) medicalization of breastfeeding. Social Science & Medicine. 2014;100:159-66.

21. Wright AI, Hurst NM. Personal Infant Feeding Experiences of Postpartum Nurses Affect How They Provide Breastfeeding Support. JOGNN - Journal of Obstetric, Gynecologic, and Neonatal Nursing. 2018;47(3):342-51.
