# Supplementary material for: Experiences that influence how trained providers support women with breastfeeding: A systematic review of qualitative evidence
Source: PLoS One. 2022 Oct 14;17(10):e0275608. doi: 10.1371/journal.pone.0275608 (PMC9565393; doi:10.1371/journal.pone.0275608)
Supplement: S3 Table — (DOCX) [file pone.0275608.s004.docx]

## S3: CERQual Evidence Profile (Lewin *et al.,* 2018)

|  | Summary of review finding | Supporting data | Studies contributing to review finding | Methodological limitations | Coherence | Adequacy | Relevance | *CERQual assessment of confidence in the evidence* | *Explanation for CERQual assessment* |
| --- | --- | --- | --- | --- | --- | --- | --- | --- | --- |
| **Analytical theme 1: A personal philosophy of breastfeeding support.** | | | | | | | | |  |
| Summary statement: The experience of having a philosophy of breastfeeding support promotes individual practice styles | | | | | | | | | |
|  | **Descriptive theme 1: Personal breastfeeding experience** | | | | | | | | |
| 1 | Trained breastfeeding support providers personal experience of breastfeeding, whether, positive or negative, influences their approach to supporting women to breastfeed. | *“I notice that I sometimes have less patience...sometimes I think please...just do it,keep going...And I sometimes say that: if I have been able to do that three times, then you can do that too I am 100% convinced of that” (Midwife)* | 12 studies –  1, 5, 6, 8, 12, 13, 14, 15, 16, 18, 19, 21 | **Minor** methodological limitations as reflexivity was not addressed in 9 of the 12 studies. The breastfeeding experience of the researchers could potentially have an influence on the interpretation of the data of other breastfeeding women. 4 studies have limited descriptions of data analysis methods (it is not clear how themes were generated). | **Minor** concerns about coherence given that in two studies (14 & 18) some health professionals felt that professional practice was distinct from any personal experience. Data varied in richness between studies. | **Minor** concerns about adequacy. There was a large quantity of data from a range of high-income countries representing a variety of breastfeeding support roles. | **No or very minor concerns** regarding relevance as the findings match the context of the review question | **High confidence** | No downgrading of overall assessment: It is highly likely that the review finding of personal breastfeeding experience is a reasonable representation of an experience which influences breastfeeding support due a data from range of studies and consistency of the data in relation to the review questions. |
|  | **Descriptive theme 2: Belief in the value and process of breastfeeding** | | | | | | | | |
| 2 | Trained breastfeeding support providers belief in the value of breastmilk and/or breastfeeding to the mother and baby dyad, and belief about how breastfeeding works influences how they supported women to breastfeed. | *Colostrum was ‘super milk’, ‘three times the strength of mature milk’, ‘undiluted cordial’, or an ‘atomic bomb’ of breastmilk (observation of midwife-woman interactions)* | 10 studies -  3, 4, 5, 7, 8, 12, 14, 16, 20, 21 | **Minor** methodological limitations as reflexivity was not addressed in 7 out of the 10 studies. This was not seen to potentially contribute to either respondent or researcher bias for this finding. 3 of the studies had methodological limitations due to data analysis methods. | **No or very minor concerns** about coherence. Although generally the case that trained breastfeeding support providers believed breastfeeding to be important and worthy of skilled support, one study reported some participants who felt that breastfeeding was a natural skill that should not require particular help, and one study reported participants who did not believe breastmilk to be more beneficial to the mother baby dyad. | **Minor** concerns about adequacy due to the number of studies with data contributing to the review finding, but the data was rich. | **No or very minor concerns** regarding relevance as the findings match the context of the review question. | **High confidence** | No downgrading of overall assessment: It is highly likely that this review finding focusing on trained breastfeeding support providers belief in the value of breastfeeding, and how breastfeeding works, is a reasonable representation of an experience which influences breastfeeding support. |
|  | **Descriptive theme 3: Knowledge for practice** | | | | | | | | |
| 3 | Trained breastfeeding support providers use a range of knowledge sources to inform their breastfeeding support practice, for example training courses, policies, observing breastfeeding, personal experiential knowledge. | *“You have got to have guidelines and you have got to have policies so that we are all saying the same thing. But you can apply them differently” (Midwife)*  *They were often happy to ‘share’ with new mothers breastfeeding tips that ‘worked for them’ (Maternal newborn nurses)* | 14 studies -  2, 3, 4, 7, 8, 10, 11, 12, 13, 14, 15, 18, 19, 21 | **Minor** methodological limitations as reflexivity was not addressed in 10 out of the 14 studies but this was not seen to be a potential threat to either respondent or researcher bias for this finding. One study was assessed as having methodological limitations related to how participants were recruited (The Head of Midwifery recruited the midwives) but this study contributed data from only 4 participants. 4 studies had methodological limitations due to lack of detailed description of the data analysis methods used in the study. | **Moderate** concerns about coherence given the range of trainings and settings of the trained breastfeeding support providers. However overall the data from these studies fits the review finding as no disconfirming cases were found where participants reported not using forms of knowledge in their practice. | **Minor** concerns  only due to supporting data from a fairly large range of studies with a variety of trained breastfeeding support providers | **No or very minor concerns** regarding relevance as the finding matches the context of the review questions. | **Moderate confidence** | It is likely that this review finding focusing on knowledge-use in practice, is a reasonable representation of an experience which influences breastfeeding support. Confidence was downgraded to moderate due to concerns about coherence as participants have undergone a range of different breastfeeding training courses. |
| **Analytical theme 2: Tensions and teamwork in practice.** | | | | | | | | | |
| Summary statement: The experience of disconnection from, or collaboration with, colleagues undermines or progresses breastfeeding support practice. | | | | | | | | | |
| **Descriptive theme 6: Collaboration** | | | | | | | | | |
| 6 | Collaboration with other trained breastfeeding supporters influenced how participants provided breastfeeding support in relation to supporting women as a team and use of referral systems. | *“I used to work with (obstetricians) where I’d say to them, “Do me a favor. Tell every mom she’s got great equipment. It’ll take you 30 seconds. It will save me 4 hours on the other end.” Lactation consultant* | 4 studies - 1, 2, 6, 11 | **Minor** methodological limitations. 3 of the 4 papers were limited in researcher reflexivity but the role of the researcher is not seen as affecting reports of collaboration. One study was limited in its detail of data analysis. | **No or very minor concerns** about coherence as in these studies there is consistent data to support the finding. | **Moderate** concerns about adequacy as only 4 out of 20 papers contributed to this finding, however the data was rich. | **Minor concerns** regarding relevance as data from the studies contributing to this finding do not include trained peer supporters and so do not completely match the population of the review question. | **Moderate confidence** | Finding has been downgraded to moderate confidence due to the concerns about adequacy as a relatively small number of studies contributed data to this finding. |
| **Descriptive theme 7 : Inconsistency in support and advice** | | | | | | | | | |
| 7 | Inconsistency in breastfeeding support provided by others influences how participants provide subsequent support. | *“I always make a point of saying to the Mums that just because I’ve said something different doesn’t mean it’s necessarily conflicting. It’s just that you’ve moved on in the plan of care” Midwife* | 6 studies -  1, 2, 6, 8, 11, 16 | **Minor** methodological limitations . 4 studies were limited in researcher reflexivity but this finding would not be affected by the researcher role. One contributing study was assessed as having methodological limitations due to potential for bias in recruitment and limited detail about data analysis. | **Minor** concerns about coherence as the data contributing to this finding were only briefly mentioned, not explored in detail in some studies. | **Moderate** concerns about adequacy as a relatively small number of studies contributed to this study but these contributed rich data. | **No or minor concerns** regarding relevance as data were from participants representation a range of participants different support-roles. | **Moderate confidence** | It is likely that this review finding is a reasonable representation of an experience which influences breastfeeding support due to data from range of studies and consistency of the data in relation to the review questions. |
| **Descriptive theme 8: Opinions of others** | | | | | | | | |  |
| 8 | When participants perceive that colleagues have certain opinions about their practice, this influences breastfeeding support provision. | *“sometimes you feel like you are being made fun of by sitting patiently with a cup trying to feed this baby. ‘‘Oh, give it a bottle!’’ other midwives say.” (Midwife)* | 6 studies -  4, 6, 8, 10, 13, 18 | **Minor** methodological limitations reflexivity was not addressed in the studies, but the researcher role was not seen to influence how participants perceived the opinion of others. One contributing study was assessed as having methodological limitations due to potential for bias in recruitment and limited detail about data analysis. | **Minor** concerns only about coherence as the data supporting this finding was mostly consistent. | **Moderate** concerns about adequacy due to the limited number of studies, however the data was sufficiently rich in these studies in relation to this finding. | **Moderate concerns** regarding relevance as data from the studies contributing to this finding do not include lactation consultants or trained peer supporters and so do not completely match the population of the review question. | **Low confidence** | Confidence in this finding was downgraded to low due to concerns about adequacy and relevance related to the number of studies and limited representation of support-role types contributing data. |
| **Analytical theme 3: Negotiating organisational constraints** | | | | | | | | | |
| **Summary statement:** The experience of negotiating organisational expectations and constraints impacts role-enactment. | | | | | | | | | |
| **Descriptive theme 9: Time and resources** | | | | | | | | | |
| 9 | Trained breastfeeding support providers reported that the reality and perception of having adequate time and resources influenced how they supported women to breastfeed. | *“they wanted folk out of labour suite and into the postnatal ward, clear the decks” (Midwife)*  *‘. . . it’s just easier and quicker to lean in over the top and to put those little babies on’ (Midwife)* | 13 studies -  1, 4, 5, 6, 7, 8, 9, 10, 11, 12, 13, 16, 18 | **Minor** methodological limitations. Researcher reflexivity was unclear in 10 of the studies but the researcher role is not seen to affect what participants would report about resources needed to enact their role. | **No or very minor** concerns about coherence as in these studies there is consistent data to support this finding. | **Minor** concerns about adequacy only as data supported this finding from 13 out of 19 papers with participants from a range of support-role types. | **Minor concerns** regarding relevance as data from the studies contributing to this finding do not include trained peer supporters and so do not completely match the population of the review question. | **High confidence** | It is highly likely that this review finding is a reasonable representation of an experience which influences breastfeeding support due to data from range of studies and consistency of the data in relation to the review questions. |
| **Descriptive theme 10: Organisational values** | | | | | | | | | |
| 10 | Participant’s perception of the priority given to breastfeeding support within their organisation influenced how they enact their role. | *“Sometimes you have to say I’ll send a midwife out, but you know that’s a resource that is precious. All you can do is make sure they’ve got a visit the next day if it’s the middle of the night. [Pause] But that’s a long gap and that’s not her answer at that time ” (Midwife)* | 4 studies - 1, 6, 8, 10 | **Minor** methodological limitations. All 4 studies lacked researcher reflexivity but is unclear whether in these studies the researcher role would affect how participants reported their perception of the values of the organisation. | **Minor concerns** about coherence as in some studies this experience of the value of breastfeeding support within the organisation was not explored in detail. | **Moderate concerns** about adequacy as only 4 out of 19 papers contributed to this finding, however the data was rich. | **Minor concerns** about relevance as data from the studies contributing to this finding do not include trained peer supporters and so do not completely match the population of the review question. | **Moderate confidence** | Confidence in this finding was downgraded to moderate due to concerns about adequacy due to the small number of studies contributing to the finding. |
| **Descriptive theme 11: Expectation of role** | | | | | | | | | |
| 11 | Participant’s expectation of what their role, situated within their organisation, entails, influenced how they supported women to breastfeed. | *“Breastfeeding shouldn’t be a hard sell...I mean my job is not to push somebody” (Maternal newborn nurse)*  *Sharon explained her job as,“to make sure that the woman who quits nursing quits because she is emotionally ready to be done with it, rather than she doesn’t know what else to do.” (Lactation consultant)* | 7 studies –  3, 8, 9, 10, 13, 16, 20 | **Minor** methodological limitations**.** 4 of the studies had methodological concerns in regard to researcher reflexivity but the researcher role was not seen as potentially influential in this finding. One study had limited detail on data analysis. | **Moderate concerns** about coherence as in some studies this experience of the value of breastfeeding support within the organisation was not explored in detail | **Moderate concerns** about adequacy as only 6 out of 19 studies contributed to this finding, however the data was rich. | **Minor concerns** about relevance as data from the studies contributing to this finding do not include trained peer supporters and so do not completely match the population of the review question. | **Low confidence** | There is low confidence in this finding due to moderate concerns about coherence and adequacy of data. |
| **Analytical Theme 4: Encounters with breastfeeding women.** | | | | | | | | | |
| Summary statement: The experience of interacting with breastfeeding women fosters a tailoring of support to women’s perceived needs | | | | | | | | | |
|  | **Descriptive theme 4: Perceptions of mothers breastfeeding reality** | | | | | | | | |
| 4 | Trained breastfeeding supporters’ perceptions of the mother’s breastfeeding intention and capability influences breastfeeding support provision. | *Midwives from the hospital setting also indicate that the hands-on approach is more often used with mothers who themselves take little initiative to put their babies on the breast and/or are less motivated to breastfeed.* | 15 studies –  1, 2, 3, 4, 5, 6, 7, 8, 9, 10, 11, 13, 16, 18,20 | **Minor** methodological limitations as reflexivity was not addressed in 12 of the 15 studies. This was not seen to be a potential threat to either respondent or researcher bias for this finding. Two of the studies contributing data had methodological limitations due to lack of detailed data analysis methods. | **Moderate** concerns about coherence that perceptions of breastfeeding reality (in this review meaning motivation and capability) may be interpreted differently by different participants. | **Moderate** concerns a although there was a relatively large number of studies contributing data to this review finding, the degree of richness in the data varied between studies. | **No or very minor concerns** regarding relevance as the context of the finding matches the context of the review question. | **Low confidence** | Finding has been downgraded to low due to concerns about coherence and adequacy of the data. |
|  | **Descriptive theme 5: Relationship and communication** | | | | | | | | |
| 5 | The nature of the relationship between participants and women, and participants communication style, influenced how breastfeeding support was practised. | *“I do not stand in the doorway and speak. I am very close . . .” (Midwife)*  *Comments such as “trust your instincts”, “whatever feels right”, “there are no hard and fast rules” and “you could try this or you could try that” were used to encourage women to find their own unique way to breastfeed (Peer support counsellors and privately practising midwives)* | 16 studies -  1, 2, 3, 4 5, 7, 10, 11, 13, 14, 15, 16, 17, 18, 20, 21 | **Moderate methodological limitations** 12 out of the 16 studies contributing data to this finding had methodological limitations concerning reflexivity but this was not seen to be a potential threat to either respondent or researcher bias for this finding. 5 studies had methodological limitations regarding details of data analysis. | **No or very minor concerns** about coherence as consistent data is found across the studies to support this review finding. | **Minor** concerns about adequacy as a large number of studies from different settings contributed to this finding but data were thin in several studies. | **No or very minor concerns** regarding relevance as the context of the finding matches the context of the review question. | **Moderate confidence** | Confidence in this finding was downgraded to moderate due to due to some methodological limitations and limited richness of data. |
