# Supplementary material for: Experiences that influence how trained providers support women with breastfeeding: A systematic review of qualitative evidence
Source: PLoS One. 2022 Oct 14;17(10):e0275608. doi: 10.1371/journal.pone.0275608 (PMC9565393; doi:10.1371/journal.pone.0275608)
Supplement: Attachment — Submitted filename: Response to Reviewers .docx [file pone.0275608.s006.docx]

Level 7, Medical Biology Centre,

Queen’s University of Belfast,

97, Lisburn Road

Belfast BT97BL

UK

Email: mchesnel01@qub.ac.uk

Associate Editor, PLOS One

Date of resubmission: 28/04/22

Dear Editor,

Thank you for reviewing our submission titled:

**Supporting breastfeeding women: a thematic synthesis of experiences which influence trained providers' practice**. PONE-D-21-32296

I am attaching a revised manuscript with agreement from the co-authors following changes suggested by the two reviewers. I would like to thank both reviewers for their helpful comments which we believe have strengthened the paper.

The request for re-submission included specific comments about content from both reviewers alongside suggestions to improve the formatting of the document. Our responses to the reviewers’ comments are provided in the table below.

We hope this has answered all of the comments made and appreciate the review of our manuscript. If further clarity is required on any aspect we are happy to address and look forward to your response on our revised version.

Yours sincerely,

Mary Jo Chesnel

| Comment | Response | Edits in Revised Manuscript |
| --- | --- | --- |
| 1. Please ensure that your manuscript meets PLOS ONE's style requirements, including those for file naming. The PLOS ONE style templates can be found at  https://journals.plos.org/plosone/s/file?id=wjVg/PLOSOne_formatting_sample_main_body.pdf and  https://journals.plos.org/plosone/s/file?id=ba62/PLOSOne_formatting_sample_title_authors_affiliations.pdf | Edited | Figures have been uploaded to PACE tool.  Unfortunately we were unable to achieve permissions for the illustrations in the graphic Figure 2 Thematic synthesis and these have been removed from the Figure. |
| 2. We noted in your submission details that a portion of your manuscript may have been presented or published elsewhere. Please clarify whether this publication was peer-reviewed and formally published. If this work was previously peer-reviewed and published, in the cover letter please provide the reason that this work does not constitute dual publication and should be included in the current manuscript. | This manuscript does not represent dual publication. Only the protocol (the planned approach that would be undertaken) for this systematic review has been published [1] | N/A |
| 3. Please note that in order to use the direct billing option the corresponding author must be affiliated with the chosen institute. Please either amend your manuscript to change the affiliation or corresponding author, or email us at plosone@plos.org with a request to remove this option. | Direct billing is not required as Queen’s University Belfast has an institutional agreement with PLOS One for publication. The corresponding author is a PhD candidate at Queen’s University and has previously published in PLOS One [1] | N/A |
| 4. Please include a caption for figure 1. | Edited as described: | Line 221: Caption included |
| 1. Is the manuscript technically sound, and do the data support the conclusions?  The manuscript must describe a technically sound piece of scientific research with data that supports the conclusions. Experiments must have been conducted rigorously, with appropriate controls, replication, and sample sizes. The conclusions must be drawn appropriately based on the data presented.  Reviewer #1: Partly  Reviewer #2: Yes | Data does support the conclusions as highlighted by reviewer #2 | N/A |
| 2. Has the statistical analysis been performed appropriately and rigorously?  Reviewer #1: No  Reviewer #2: N/A | N/A as per Reviewer 2 | N/A |
| 3. Have the authors made all data underlying the findings in their manuscript fully available?  The PLOS Data policy requires authors to make all data underlying the findings described in their manuscript fully available without restriction, with rare exception (please refer to the Data Availability Statement in the manuscript PDF file). The data should be provided as part of the manuscript or its supporting information, or deposited to a public repository. For example, in addition to summary statistics, the data points behind means, medians and variance measures should be available. If there are restrictions on publicly sharing data—e.g. participant privacy or use of data from a third party—those must be specified.  Reviewer #1: No  Reviewer #2: Yes | Yes, data underlying the findings is fully available as highlighted by Reviewer #2. This manuscript is a systematic review of qualitative evidence and the published papers from which the data have been extracted are referenced. | N/A |
| 4. Is the manuscript presented in an intelligible fashion and written in standard English?  PLOS ONE does not copyedit accepted manuscripts, so the language in submitted articles must be clear, correct, and unambiguous. Any typographical or grammatical errors should be corrected at revision, so please note any specific errors here.  Reviewer #1: Yes  Reviewer #2: Yes | Edited | Proof reading carefully undertaken and edits of typographical and grammatical errors amended |
| Reviewer #1: This work is novel and raises relevant public health issue. Structure of the paper is well established. There is a clear aim of the study. I found that you missed PRISMA flow chart with systematic search criteria, inclusion and exclusion criteria in the figure. Please do not start sentence with number (eg line 37). | Thank you for your comments, I have addressed the issues as follows: | Line 219: PRISMA reference included  Line 221: Figure of PRISMA flow chart included in text (Figure 1). Inclusion and exclusion criteria now included in the PRISMA flow chart figure.  Line 163-164: The reader is directed to the supplementary file S1 Table of Search histories.  Lines 131-151: Now includes Inclusion & Exclusion Criteria  Line 41: Edited to read ‘This systematic review includes 21 papers…’ |
| Reviewer #2: The methodology of this systematic review is well conducted and reported. It also covers an important area of breast feeding which demands in-depth understanding through qualitative research. The necessary supporting information for different steps of systematic review is provided in the supplemental file. However, discussion section could have been richer.  Following comments could help improve the quality of this manuscript.  1. Title: It is better to clearly specify that it is a systematic review of qualitative research or evidence in the title. The current title doesn’t clearly give that message to the readers | Thank you for your comments. I have changed the title in response and will detail the changes made to the discussion section later in this table of edits. | Line 1: Title changed and now reads ‘Experiences that influence how trained providers support women with breastfeeding: a systematic review of qualitative evidence’ |
| 2. Abstract:  Findings- Please don’t start the sentence with number.  Please also specify the total search results out of which the 21 papers were included. | Edited as requested | Line 41: The sentence no longer starts with a number, it now reads: This systematic review includes 21 papers comprising 368 participants from the 1811 records retrieved. |
| 3. Methods  -Please begin this section by specifying that you have followed PRISMA guidelines,  and also provide details of protocol registration as mentioned in the abstract. | Edited as requested | Line 106-108: This sentence now reads: PRISMA guidelines were followed in conducting this review [2]. The review protocol is registered in the International Prospective Register of Systematic Reviews: PROSPERO registration number CRD42020207380 and has been published. |
| -Search strategy – line 123 – Please clearly specify what your PEOT is by elaborating criteria for each category. | Edited and now included.  In addition this information is included in the Supplementary Information file S1 Table of Search histories | Line 124-128: This section specifies the PEOT and now reads: A systematic search strategy was developed in collaboration with an expert subject librarian using a PEOT [27] question format: Population (trained breastfeeding support providers as per study definition above), Exposure (breastfeeding and breastfeeding support provision), Outcome (experiences that influence breastfeeding support practices), Type of study (studies that have qualitative methods or findings).  Line 163-164 now reads: The search strategy is given in the supplementary file S1 Table of search histories. |
| -Though the authors have mentioned the inclusion and exclusion criteria, it is better to present it in a more structured way sooner so that the readers don’t have to search through the text to figure it out. | Edited so that inclusion and exclusion criteria are presented sooner, following PEOT information. A brief summary of the inclusion and exclusion criteria has also been included in the PRISMA flow chart. | Lines 131- 164: Inclusion and Exclusion criteria presented to reader sooner in the manuscript  Line 221: Figure 1 PRISMA flow chart contains a brief summary of the inclusion and exclusion criteria |
| 4. Findings- please cite the flow diagram of the findings described in the main text. | Edited as detailed in the next column. | Line 219: PRISMA referenced  Line 221: PRISMA figure cited |
| -Study characteristics- please break the paragraph. | Edited. The paragraph has been split into two smaller paragraphs with the first focusing on study characteristics and the second focusing on the study quality | Line 236-255: Study characteristics section  Line 256-263: Critical appraisal section |
| -Pls, dedicate a paragraph to describe PRISMA flow chart and another paragraph describing the study characteristics and describe the table on characteristic of studies. | Edited as detailed in the next column. | Lines 214- 220: Paragraph describes the PRISMA flow chart  Lines 236-255: Paragraph describes the study characteristics.  Lines 228-239 describe the table of study characteristics and the first sentence now reads: Table 1 describes data extracted from each study and the study quality under the headings Title, Author, Year, Country, Study aim or research question, Practice setting, Methodology and method, Population and sample size, COREQ score and Findings (Themes/subthemes).  Lines172-179 also describe the data extraction form and study characteristics of interest. |
| -Pls also cite the table that is included in the supplemental file.  -Technically, that table should be part of main text rather than a table in supplemental file. | Edited and the Study characteristics table has been moved into main text as requested t rather than supplemental file and cited. | Line 228-230: Now cites Table 1 Study characteristics in text  Line 225: Now contains Table 1 Study characteristics. |
| -The PRISMA flow chart should also be part of the main text. | Edited as requested. PRISMA flow chart moved into main text as figure 1. | Line 221 will now contain PRISMA flow chart in text as Figure 1 |
| -Line 241- Subheading “Findings” here makes no sense. Please rename with what this subsection represents. Probably the themes? | Edited and renamed as Themes as requested | Line 256 edited to read ‘Themes’ |
| 5. Discussion  Discussion section needs a bit of restructuring. This is just a suggestion; the flow seems a bit interrupted which could be improved. For example, it could make sense to discuss the research questions before going to each theme. | Thank you for your comment, The discussion has been edited, restructured and additional content included as follows: | Lines 422-582: The discussion has been restructured and now begins with a discussion of the research questions as requested. |
| Also the paragraphs elaborating on themes seems simplistic. It doesn’t provide as much depth as it demands. While the authors have compared and contrasted the findings of each theme with other studies, it would be better if the authors could also provide deeper insights into the “why” and “how” for each theme and what the authors think can be done to improve things under each theme. | Thank you for these suggestions. The discussion has been revised as follows: | The discussion has been enhanced with revisions to the theme sections in lines 483-567 and inclusion of additional references [3-6].  In each paragraph discussing a theme (within this discussion section) the second sentence summarises improvements to be made in practice in relation to each theme, and then further insights are developed for the remainder of each paragraph, for example lines 530-533.  In addition, lines 586-602 now include three examples for practice improvement. |
| There are typing errors at some places. Please thoroughly proofread the manuscript before submitting the revised version. | Edited  Supplementary information files have been re-numbered due to some information now being presented as Figures/ Tables instead of supplementary information. The PRISMA 2020 checklist was edited to reflect the page numbers in the revised manuscript. | Supplementary Information now ordered as follows:  S1 Table of Search histories  S2 Prevalence of themes  S3 CERQual Evidence table  S4 COREQ Appraisal of Reporting  S5 PRISMA 2020 Checklist |
|  |  |  |

1.	Chesnel M, Healy M, McNeill J. The experiences of trained breastfeeding support providers that influence how breastfeeding support is practised: A protocol for a qualitative evidence synthesis. PLOS One. 2021;16(7):e0254445.

2.	Page M, McKenzie J, Bossuyt P, Boutron I, Hoffmann T, Mulrow C, et al. The PRISMA 2020 statement: an updated guideline for reporting systematic reviews. BMJ. 2021;372.

3.	Prussing E, Browne G, Dowse E, Wilson A. Implementing midwifery continuity of care models in NSW regional areas. Women and Birth. 2018;31:S5-S6.

4.	Brown A, Chucha S, Trickey H. Becoming breastfeeding friendly in Wales: Recommendations for scaling up breastfeeding support. Maternal & Child Nutrition. 2022:e13355.

5.	Chang Y-S, Beake MS, Kam MJ, Lok KY-W, Bick D. Views and experiences of women, peer supporters and healthcare professionals on breastfeeding peer support: A systematic review of qualitative studies. Midwifery. 2022:103299.

6.	Perez-Escamilla R, Chapman DJ. Breastfeeding protection, promotion, and support in the United States: a time to nudge, a time to measure. J Hum Lact. 2012;28(2):118-21.
