# Supplementary material for: Experiences that influence how trained providers support women with breastfeeding: A systematic review of qualitative evidence
Source: PLoS One. 2022 Oct 14;17(10):e0275608. doi: 10.1371/journal.pone.0275608 (PMC9565393; doi:10.1371/journal.pone.0275608)
Supplement: Attachment — Submitted filename: Response to reviewers.docx [file pone.0275608.s007.docx]

Level 7, Medical Biology Centre,

Queen’s University of Belfast,

97, Lisburn Road

Belfast BT97BL

UK

Email: mchesnel01@qub.ac.uk

Associate Editor, PLOS One

Date of resubmission: 17/09/22

Dear Editor,

Thank you for reviewing our submission titled *“Experiences that influence how trained providers support women with breastfeeding: A systematic review of qualitative evidence.”* I am attaching a revised manuscript updated with the minor revisions advised and would like to thank the reviewers for their helpful suggestions which we believe has strengthened the paper.

The request for re-submission included suggested improvements in the formatting of the document and specific comments. Detail in relation to these requirements from the reviewers is provided in the table below.

We hope this has answered all of the comments received and appreciate the review of our paper. If further clarity is required on any aspect we are happy to address and look forward to your response on our revised version.

Yours sincerely

Mary Jo Chesnel

| **Reviewer comment** | **Author response** | **Revised manuscript now reads**  **(line numbering of revised track changes document)** |
| --- | --- | --- |
|  |  |  |
| **Abstract**  Line 45-46 findings –is it 1811 record screened or retrieved? Perhaps screened would be the more appropriate term | Edited to reflect the number of records screened by title and abstract. Of note, 1811 records were identified and duplicates were removed by Covidence software leaving 977 for screening. | Line 44-45: “977 records were screened” |
| It is also better to report the impression of quality assessment/ risk of bias assessment of the included studies.  Pls refer to PRISMA 2020 checklists for abstract  https://prisma-statement.org//documents/PRISMA_2020_abstract_checklist.pdf to ensure all the information needed to be reported for the abstract are included | The PRISMA abstract has been reviewed and the abstract complies with all elements with the exception of the requirement for funding information which is provided in the financial disclosure statement of the PLOS submission.  The abstract has been reworded slightly to ensure the PRISMA abstract checklist elements are included within the limits of the PLOS abstract word limit. | Line 35: “including Medline and CINAHL”  Line 36-39: “Studies eligible for inclusion reported professional and trained peer experiences of supporting women to breastfeed. PRISMA guidelines were followed and included studies were quality appraised using the CASP Qualitative Checklist.”  Line 46: “Following quality appraisal, all studies were deemed suitable for inclusion”. |
| **Methods**  For systematic review of qualitative studies, we focus on PICo which stands for population, interest, and context as the outcomes are not directly measurable in qualitative studies unlike for quantitative studies. The term “Outcome” could be replaced with Context instead. E.g search strategy Lines 136-138 and Data Extraction Line 215 of the tack changes file. | Edited with suggestion to replace outcome with context – thank you for this suggestion. This necessitated a slight restructuring of the sentence, and replacement of the word “Outcome” with “Context” in Supplementary material S1 (Table of search histories) and Figure 1 (PRISMA flow diagram), and removal of the word “outcomes” from line 181 to ensure consistency throughout the manuscript. | Lines 128-130: “A systematic search strategy was developed in collaboration with an expert subject librarian guided by a PEOT [27] format. For the purposes of this review “context” replaces “Outcome” in the mnemonic as outcomes are not directly measurable in qualitative studies.”  Line 181: “Meaningful sections of text were extracted that identified experiences...” |
| **Findings**  Included studies – as per the PRISMA checklist, it is also important to mention the reasons for exclusion for the studies excluded after full-text screening. | Edited to include a sentence stating reasons for exclusion as illustrated in Figure 1 PRISMA flow chart. | Line 223-226: “25 were excluded for reasons of wrong population (e.g. not providing routine breastfeeding support), wrong exposure (no data on breastfeeding or breastfeeding support), wrong context (no data on breastfeeding support practices) or wrong type of study (e.g. pilot evaluations).” |
| The tables should follow the description (text) and not precede it. Pls re-order the description of Table 1 followed by the Table placed below it. | Edited so that the description precedes the table. Also text omitted in error (theme names in table) has now been included. | Line 248-251: “Data was extracted from each study comprising Title, Author, Year, Country, Study aim or research question, Practice setting, Methodology and method, Population and sample size, COREQ score and Findings (Themes/subthemes). Study characteristics and quality appraisal scores are summarized in Table 1.” |
| **Critical appraisal**  Line 292- Please provide the full name of the tool. CASP has different tools for different study designs. So please specify it as the CASP Qualitative Studies Checklist if that was the tool used.  The critical appraisal in the results section should elaborate on the overall impression based on CERQual Evidence Profile and COREQ. What were the areas that were not reported by most studies? How many studies showed low and moderate confidence and what they lacked in their methods which lead the studies to be rated as moderate or low? Also what were the strengths of most studies in terms of critical appraisal? | Edited as advised.  Edited to explain the overall impression of study quality. Further information has been included to demonstrate how the quality appraisal based on CASP and COREQ informed the CERQual Evidence Profile of confidence in this reviews’ findings. This explanation of confidence in our findings, informed by prior quality appraisal of the contributing studies, has been added further into the findings section as it relates to the overall research output of this review. | Line 277: “The Critical Appraisal Skills Programme (CASP) Qualitative Checklist [32] was used…”  Lines 281-282: “Most studies did not report on researcher reflexivity. Description of analytical methods was limited in five of the studies.”  Lines 285-286: “Overall, strengths of the studies lay in the congruence of the research aims and objectives with the study design.”  Lines 297-303: “Confidence in the review findings was assessed using the CERQual tool [34]. There was high confidence in three of the descriptive themes. Confidence was downgraded to moderate (five descriptive themes) or low (three descriptive themes) when there was concern about any of the four components of the CERQual assessment [34]: methodological limitations, coherence, adequacy of data, and relevance as reported in supplementary information S3.” |
| **Limitations**  Limitations need to be elaborated. As per the PRISMA the limitations can be divided into two parts. The first part should focus on the limitations within the included studies such as their methodological biases, overall impression from critical appraisal. What the studies lacked which may have affected the interpretation in this systematic review? There seemed to be a few studies that showed low to moderate confidence in CERQual Evidence Profile, which need to be addressed as limitation of the included studies. Also not all studies fulfilled all the reported criteria as per COREQ.  The second part should focus on the limitations that may have arisen during the review method such as limitations in search, inclusion, language bias (as you’ve only included English studies), etc. | The strengths and limitations section has been edited, firstly, to highlight methodological limitations within the included studies. Secondly, the CERQual evidence profile provides a measure of confidence in the findings of the review itself once the thematic synthesis is complete, to enable transparency of the review findings for the reader. Limitations in the review are now stated in terms of confidence in findings as per CERQual assessment, the limited representations of certain roles, and limits in search including language and publication date limiters. | Lines 653-666: “Quality appraisal of the studies, data analysis, theme development and application of the CERQual tool have been described in detail in the text and supplementary material, increasing transparency for the reader and trustworthiness of the review. There was high confidence in the descriptive themes *Personal breastfeeding experience, Belief in the value and process of breastfeeding* and *Time and resources* due to the adequacy of relevant coherent data and only minor concerns about possible methodological bias.  Methodological limitations within the studies include lack of transparency in reporting, as no study fulfilled all of the COREQ reporting criteria, with researcher reflexivity under-reported in most studies. Limitations of the review findings in their entirety were assessed using the CERQual tool. Concern about inadequate, irrelevant or incoherent data resulted in the downgrading of some of this review’s descriptive themes from high confidence to moderate or low confidence in findings. There is a paucity of general practitioner, health visitor and trained peer supporter representation in the studies…”  Line 671-675: “Heterogeneity of methodologies, population demographics and the settings of breastfeeding support provision represented in this review has limited synthesis and as such the wider transferability of findings.  Despite conducting an extensive database search it is possible that not all relevant records were retrieved due to the use of search limiters such as English language and publication date.” |
|  |  |  |
